# From Fiber Architecture to Digital Twins: Characterization, Orientation Modeling, and Artificial Intelligence in Short Fiber-Reinforced Polymer Composites

**DOI:** 10.3390/polym18202589

**Published:** 2026-10-21

**Authors:** Ion-Dragoș Uțu, Diana Uțu

**Affiliations:** 1 Department of Materials and Fabrication Engineering, Politehnica University Timisoara, Bulevardul Mihai Viteazul No. 1, 300222 Timișoara, Romania; dragos.utu@upt.ro; 2 Department II, Pharmacology, Physiology and Pathophysiology, Faculty of Pharmacy, Victor Babeș University of Medicine and Pharmacy Timișoara, 2 Eftimie Murgu Square, 300041 Timișoara, Romania

**Keywords:** short fiber-reinforced polymer composites, fiber orientation, injection molding, X-ray micro-computed tomography, orientation tensor, multiscale modeling, artificial intelligence, machine learning, physics-informed neural networks, digital twin

## Abstract

Short fiber-reinforced polymer composites (SFRPCs) are widely used in lightweight components because they combine low density, good mechanical performance, and high-volume processability. Their local properties, however, are governed by a fiber architecture—fiber length, orientation, dispersion, and defects—that is created during processing and varies within each molded part. This review examines how this architecture can be measured, predicted, and carried into digital representations of the manufacturing process. It covers the mechanisms that form the orientation state during injection molding; two- and three-dimensional characterization, with emphasis on X-ray micro-computed tomography; orientation models from the Jeffery and Folgar–Tucker equations to anisotropic rotary diffusion and slow-kinetics formulations; and multiscale homogenization. Applications of artificial intelligence are then assessed for image segmentation, orientation prediction, micromechanical surrogates, and physics-informed learning. Finally, published Digital Twin implementations for composite and polymer manufacturing are classified by their level of data integration, and a layered architecture for SFRPCs is proposed. Most existing systems are digital models or digital shadows rather than bidirectionally coupled twins. The main obstacles are the scarcity of paired process–microstructure–property datasets, the lack of uncertainty propagation across scales, and the limited transferability of data-driven models.

## 1. Introduction

Short fiber-reinforced polymer composites (SFRPCs), in which discontinuous glass, carbon, or natural fibers are dispersed in a thermoplastic or thermoset matrix, occupy an important position between unreinforced polymers and continuous-fiber composites. They can be processed by injection molding, extrusion, or compression molding with cycle times of seconds to minutes, which makes them the default choice for structural and semi-structural parts produced in large series in the automotive, electrical, and consumer-goods sectors [1,2]. The price of this processability is that the reinforcement architecture is not designed but emerges from the process. During mold filling, fibers rotate, interact with one another, and break; the resulting orientation and length distributions vary from point to point within the part and through its thickness [3,4,5]. Because stiffness, strength, thermal expansion, and fatigue resistance depend strongly on this local architecture [6,7,8,9], the same material can behave very differently in two parts—or in two regions of one part—molded under different conditions.

Predicting component performance therefore requires a chain of models that connects processing conditions to the local microstructure, and the microstructure to the structural response. Such integrative process–structure–property simulation chains have been developed over the last three decades: mold-filling simulation provides the flow kinematics, an orientation model predicts the orientation tensor field, a micromechanical model converts orientation into local anisotropic stiffness, and a structural finite-element analysis evaluates the part [10,11,12,13]. Each link of this chain carries assumptions and empirical parameters, and the accuracy of the chain as a whole can only be assessed against measured microstructures. X-ray micro-computed tomography (µCT) has made such measurements increasingly routine, providing three-dimensional orientation, length, and void data at voxel sizes of about one micrometer [14,15].

Two developments have changed the outlook in recent years. First, machine learning (ML) has been applied to nearly every link of the chain: to segment fibers in µCT volumes [16,17], to replace orientation simulations with fast surrogates [18], to calibrate orientation-model parameters [19], and to accelerate micromechanical homogenization [20,21]. Second, the Digital Twin (DT) concept—a virtual representation kept synchronized with a specific physical asset through data exchange—has been proposed as a framework for integrating these elements with process monitoring [22,23,24]. For SFRPCs, the concept is particularly attractive because the most important state variable, the internal fiber architecture, cannot be observed during production and must be estimated from models calibrated with offline measurements.

### 1.1. Positioning Relative to Existing Reviews

**Figure 1:**
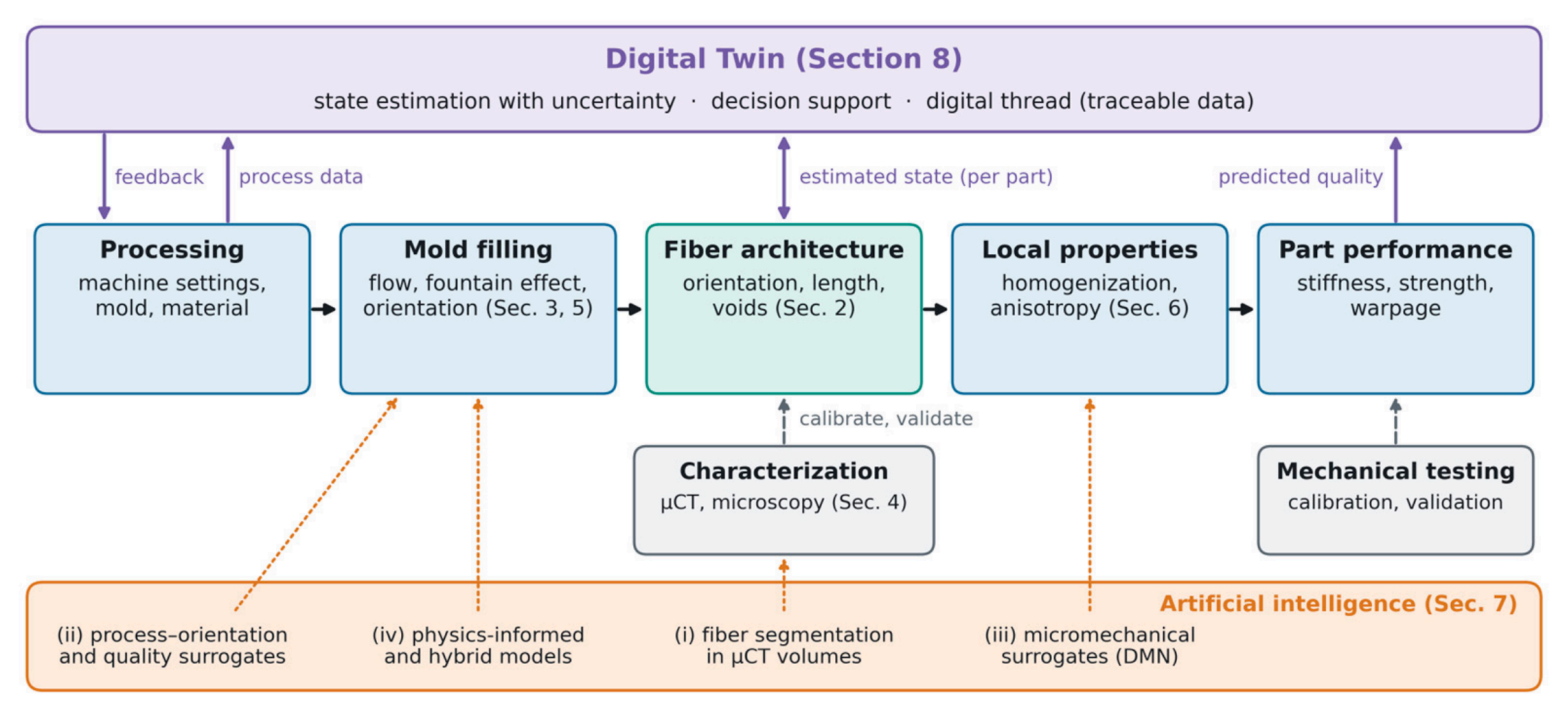
Process–structure–property chain for short fiber-reinforced polymer composites and the role of characterization, modeling, and artificial intelligence at each link. The Digital Twin couples the chain to the physical process through measured data and closes it through feedback to the process settings. Section numbers refer to the present review. Section 1 (Introduction, including the positioning of the review and the literature search) and Section 9 and Section 10 (challenges, research roadmap, and conclusions) frame the review and are not links of the chain; they are therefore not shown.

Existing reviews treat these topics largely separately or integrate only parts of the chain (Table 1): fiber-orientation models [5,12], X-ray tomography of composites [14,25], machine learning for the optimization of composite manufacturing processes [26], and Digital Twins in manufacturing [27,28]. The present review connects them. Its guiding question is which information about the fiber architecture must be measured, modeled, or learned—and with what accuracy—for a Digital Twin of an SFRPC manufacturing process to be useful. The review follows the process–structure–property chain shown in Figure 1: Section 2 defines the fiber-architecture descriptors that serve as state variables; Section 3 summarizes how the orientation state develops during injection molding; Section 4 evaluates experimental characterization methods; Section 5 reviews orientation models; Section 6 discusses multiscale homogenization; Section 7 assesses applications of artificial intelligence (AI); Section 8 classifies existing Digital Twin implementations and proposes an architecture for SFRPCs; and Section 9 identifies open challenges and proposes a research roadmap.

Several earlier reviews and integrative studies cover more than one link of this chain, and the present review should be read against them (Table 1). Tucker [5] and Kugler et al. [12] treat orientation models in depth, and Baum et al. [29] review numerical approaches for simulating the filling phase, which supplies the flow kinematics for orientation prediction; their focus is the process–orientation link. Garcea et al. [14] and Maire and Withers [25] review X-ray tomography and its quantitative analysis, with only brief consideration of how the data enter process models. Görthofer et al. [13] developed and validated a complete virtual process chain for sheet molding compound—the most integrated treatment of the chain so far—but without data-driven components or a Digital Twin perspective. Sharma et al. [30] and Malashin et al. [26] review machine learning for polymer composites and for composite processes, while Jones et al. [27], Tao et al. [28], and the U.S. National Academies [24] review Digital Twins in general, without reference to the internal microstructure of molded parts. Kalidindi et al. [31] formulated the concept of a materials digital twin, but not for SFRPC processing. The specific contribution of the present review is three-fold: (i) it treats the fiber architecture—orientation and length distributions, local fiber content, and defects—explicitly as the latent state of a manufacturing Digital Twin, which cannot be measured inline and must therefore be estimated; (ii) it assesses characterization methods, orientation models, homogenization schemes, and AI methods by what they contribute to estimating this state and its uncertainty, and it classifies existing implementations by their level of data integration (Section 8.2); and (iii) it proposes a layered architecture with an explicit state-estimation layer, positions it relative to established reference architectures, and specifies candidate estimation methods, a VVUQ framework, and a research roadmap (Section 8.3, Section 8.4, Section 8.5, Section 8.6, Section 8.7, Section 8.8, Section 8.9 and Section 9).

### 1.2. Literature Search, Selection, and Quality Assessment

This is a narrative, critical review rather than a systematic one. Literature was identified in Web of Science, Scopus, and Google Scholar using combinations of the terms “short fiber”, “fiber orientation”, “injection molding”, “micro-computed tomography”, “orientation tensor”, “machine learning”, “deep learning”, “physics-informed”, “surrogate”, and “digital twin”, complemented by backward and forward citation searches from key papers. Foundational papers were included irrespective of their publication date; for AI and Digital Twin topics, emphasis was placed on work published since 2018 that deals specifically with composite materials or polymer processing. Priority was given to studies in which predictions were compared with measured microstructures or properties.

To make the selection transparent, the search was organized in five thematic blocks (A–E) that correspond to the links of the chain, each combined with a material block; the search terms and time coverage are summarized in Table 2. Web of Science Core Collection and Scopus were searched in titles, abstracts, and keywords, and Google Scholar was used mainly for backward and forward citation searches and for conference proceedings and reports; the searches were last updated in October 2026. For blocks D (AI/ML) and E (Digital Twins), the time window was 2018–2026, whereas foundational work in blocks A–C was included irrespective of its publication date. Records were screened first by title and abstract and then by full text. Studies were included if they (i) dealt with short or discontinuous fiber-reinforced polymers or, for blocks D and E, with composite or polymer manufacturing more generally; (ii) reported a method, model, dataset, or implementation relevant to at least one link of the chain; and (iii) were published in peer-reviewed journals or proceedings, or as authoritative books, standards, and reports. Studies on continuous-fiber composites and on closely related manufacturing processes were included only when they provided methods transferable to SFRPCs, such as image segmentation, data assimilation, or process Digital Twins; conceptual Digital Twin papers without a composite or polymer application were included only when they provided definitions or reference architectures.

The quality of the included studies was appraised qualitatively with four questions: whether predictions were compared with independent measurements (validation); whether data sources, sample sizes, and analysis parameters were reported (transparency); whether uncertainties or error measures were given (uncertainty); and whether the method was applied to more than one material, geometry, or process condition (generality). These criteria were not used to exclude studies but to weight their conclusions: the statements of this review rely preferentially on studies that satisfy the first three criteria, and the limitations of individual studies are reported in the corresponding sections and tables. Because the review is narrative rather than systematic, no claim of exhaustiveness is made; statements about the absence of particular developments refer to the literature identified with this protocol up to its last update.

## 2. Fiber Architecture as a State Variable

### 2.1. Fiber Type and Geometry

Glass fibers dominate the SFRPC market because of their favorable balance of stiffness, strength, and cost; carbon fibers are used where higher specific stiffness or electrical conductivity is required; and natural fibers such as flax, hemp, or cellulose are increasingly used for their low density and renewable origin, although their lower thermal stability and moisture sensitivity restrict the processing window [1,32,33]. Typical fiber diameters are 10–17 µm for glass and 5–8 µm for carbon fibers [1], while fiber lengths in molded parts range from about 0.1 to 1 mm in short-fiber compounds and reach several millimeters in long-fiber thermoplastics [7,34].

For modeling purposes, the fiber type determines the constituent properties—modulus, strength, and density—and the fiber geometry determines how efficiently these properties are used. The aspect ratio *L*/*D* controls both the stress transfer from the matrix to the fiber and the rotational dynamics of the fiber in flow. Shear-lag analysis shows that a fiber carries its full load only over the part of its length that exceeds the critical length, so that fibers shorter than this length contribute much less to strength than their volume fraction would suggest [1,6]. Because the critical length depends on the interfacial shear strength, geometry and interface cannot be treated independently (Section 2.2). Beyond length and diameter, fiber curvature, clustering, and local variations in fiber volume fraction influence both processing and properties, but these descriptors are rarely included in process simulations (Table 3).

Natural fibers are considered in this review mainly as constituents and as a characterization challenge. Their irregular and hollow cross-sections, bundle structure, flexibility, and moisture-dependent properties violate several assumptions of the rigid, straight, cylindrical fibers on which the orientation and homogenization models of Section 5 and Section 6 are based, and validated process–structure–property chains for injection-molded natural-fiber compounds are scarce [32,35]. The modeling sections therefore refer primarily to glass and carbon fibers. For natural fibers, the same framework applies in principle, but fiber geometry and properties should enter as distributions with larger uncertainties, and the validity of each model must be established case by case (Section 6.4).

### 2.2. Fiber–Matrix Interface

Load transfer between the matrix and the fibers takes place through the interface, whose strength depends on the fiber surface chemistry, the sizing, the matrix, and the processing history. For glass fibers, silane-based sizings provide chemical coupling and are tailored to specific matrices [36]. Carbon fibers are usually surface-oxidized and sized, whereas natural fibers require compatibilizers or surface treatments because of their hydrophilic character [32,33]. The interfacial shear strength (IFSS) is commonly measured by single-fiber pull-out, microbond, or fragmentation tests [37]. In process–property simulation chains, the interface usually enters only implicitly—through a perfect-bonding assumption in homogenization models or through calibrated strength parameters—so that its variability is one of the least quantified sources of uncertainty in property predictions.

### 2.3. Processing-Induced Fiber Attrition

Fiber breakage occurs mainly during compounding and plasticization, where fibers are exposed to high stresses in the screw channels and during melting of the solid bed, and to a lesser extent during mold filling [34,38]. The final length distribution therefore differs substantially from the nominal one and varies within the part. Two modeling approaches have been established. Durin et al. described fiber breakage in twin-screw extrusion with a matrix formulation that maps the length distribution before and after each screw element [38]. Phelps et al. proposed a model that tracks a discrete fiber-length distribution at each node of a mold-filling simulation, with breakage rates derived from hydrodynamic fiber buckling, so that the length distribution can be predicted as a field together with the orientation [34]. Because the length distribution affects both orientation kinetics and mechanical properties, attrition models are an important but rarely validated link in the process chain. µCT-based length measurements, which avoid the burn-off and dispersion steps of conventional methods, are increasingly used for such validation [15].

### 2.4. Descriptors for a Digital Representation

The descriptors discussed above can be grouped according to how they enter a digital representation (Table 3). Constituent properties are inputs that remain constant during processing. Geometric descriptors such as the orientation and length distributions are outputs of the process that become inputs to structural analysis; they are the natural state variables of an SFRPC Digital Twin. Defect-related descriptors—voids, weld lines, and fiber clusters—are also process outputs, but they are rarely predicted by process simulation and are usually available only from measurements. This distinction determines which quantities a Digital Twin can predict, which it must measure, and which it can only estimate with uncertainty.

**Table 3:** Fiber-architecture descriptors of SFRPCs and their availability for a digital representation.

Descriptor	Main Influence on Properties	How It Is Measured	How It Is Predicted	Role in a Digital Twin
Fiber orientation (**a**, 𝔸)	Anisotropic stiffness and strength, thermal expansion, shrinkage, warpage	Optical method of ellipses; µCT [4,39,40]	Folgar–Tucker, ARD, RSC/RPR models (Section 5)	Primary state variable
Fiber length distribution	Reinforcement efficiency, strength, impact resistance	Burn-off and image analysis; µCT [15]	Attrition models [34,38]	State variable (often assumed constant)
Local fiber volume fraction	Local stiffness; fiber-depleted zones near walls and weld lines	µCT [14]	Rarely predicted	Measured correction field
Fiber curvature	Reduced effective stiffness; orientation kinetics	µCT with individual-fiber segmentation [15]	Not predicted by standard solvers	Measured or statistical parameter
Voids and weld lines	Strength; fatigue crack initiation	µCT; optical inspection [14]	Weld-line position from filling simulation; void content rarely	Defect map from measurement
Fiber–matrix interface (IFSS)	Strength, damage, fatigue	Pull-out, microbond, fragmentation tests [37]	Not predicted; calibrated	Uncertain parameter

### 2.5. Hybrid and Recycled Fiber Systems

Two classes of SFRPCs deserve separate mention because they modify the fiber architecture in ways that standard models do not capture. In hybrid short-fiber systems, such as polypropylene reinforced with both short glass and short carbon fibers, the two fiber populations differ in diameter, stiffness, failure strain, and length after processing. Their tensile properties deviate from a simple rule of hybrid mixtures, and the deviation (the hybrid effect) depends on the relative fiber fractions and on the length and orientation distributions of each population [41]. During loading, the fibers with the lower failure strain (carbon) break first, and the surviving fibers (glass) must bridge the resulting damage, so that dispersion and the critical lengths of both populations control the fracture behavior [42]. For modeling, each population requires its own orientation and length descriptors, and the orientation kinetics may differ between the populations because of their different aspect ratios; length-dependent orientation closures, which account for longer fibers being more strongly aligned than shorter ones, provide a framework for this case [43]. Recycled fibers—for example, carbon fibers reclaimed by pyrolysis or solvolysis—usually enter compounding with a broad length distribution, variable or missing sizing, and surface damage [44]; in injection-molded compounds of recycled carbon fibers, mean fiber lengths of only about 0.20–0.32 mm have been reported after processing [45]. For both hybrid and recycled systems, the fiber–matrix interface and the fracture behavior are less predictable than for virgin single-fiber compounds, which increases the weight of measured length and interface data in any digital representation.

## 3. Development of Fiber Orientation During Injection Molding

### 3.1. Mechanisms

Fiber orientation in a flowing melt results from three groups of mechanisms. Hydrodynamic forces rotate each fiber according to the local velocity gradient: in simple shear, an isolated rigid fiber in a Newtonian fluid follows periodic Jeffery orbits and spends most of its time nearly aligned with the flow direction, whereas in extensional flow it aligns with the stretching direction [5,46]. Fiber–fiber interactions, both hydrodynamic and mechanical, disturb these trajectories and act as a randomizing mechanism; in concentrated suspensions they limit the degree of alignment reached at steady state, which is why interaction terms are needed in all practical orientation models [47,48]. Finally, the orientation of concentrated suspensions evolves more slowly with strain than the Jeffery kinematics predict. This slow-kinetics effect has been documented in transient rheological experiments [49,50]. The three mechanisms correspond directly to the terms of the orientation models discussed in Section 5.

### 3.2. Fountain Flow and the Layered Orientation Structure

**Figure 2:**
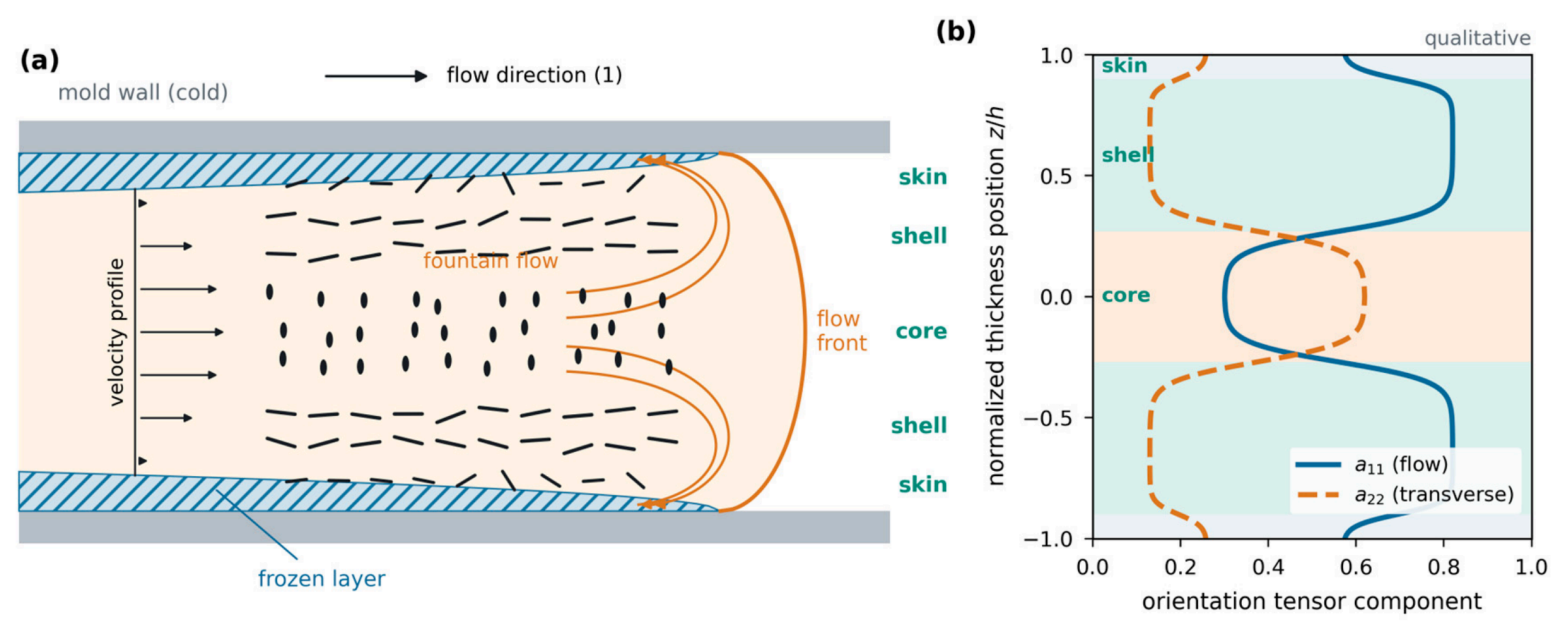
Formation of the layered fiber orientation structure in injection molding (schematic). (**a**) Fountain flow at the advancing melt front transports material from the channel center to the mold walls, where a frozen layer grows. (**b**) Typical through-thickness profiles of the orientation tensor components *a*_11_ (flow direction) and *a*_22_ (transverse direction), showing shell layers aligned with the flow and a core with transverse orientation, as observed in diverging flows. The profiles are qualitative; their actual shape depends on the material, geometry, and processing conditions.

In a thin cavity, the melt advances with a fountain flow: material from the center of the channel moves toward the flow front, where it is deflected toward the cold mold walls and deposited there [51]. Together with the through-thickness distribution of the shear rate, this produces the characteristic layered orientation structure of injection-molded SFRPCs shown in Figure 2 [4,5]. In the shell layers, located between the walls and the mid-plane, high shear rates align the fibers predominantly with the flow direction. In the core, the shear rate is low and the orientation is governed by the in-plane velocity gradients; in diverging flows, such as those near a center gate, stretching in the transverse direction orients the core fibers perpendicular to the main flow. Adjacent to the walls, a thin skin layer is formed from material that was deposited by the fountain flow and frozen rapidly; its orientation is often less aligned than that of the shell [4,5]. The relative thickness of the core depends on the material, the processing conditions, and the part geometry. It is a sensitive indicator of the predictive quality of orientation models, which tend to underestimate it when slow kinetics, flow–fiber coupling, or the low-shear-rate rheology of the suspension are neglected [52,53].

The three-layer picture is a useful idealization rather than a sharp partition. µCT studies show gradual transitions between the layers, asymmetries through the thickness, and additional in-plane variations near gates, ribs, and weld lines [40,54,55]. For modeling purposes, the layered structure is therefore best treated as a continuous through-thickness orientation field rather than as a stack of homogeneous laminae.

### 3.3. Influence of Processing Conditions and Part Geometry

Processing conditions act on orientation mainly through the flow kinematics and the viscosity field (Table 4). Higher injection speeds increase the shear rates and generally widen the shell layers relative to the core, although the net effect also depends on the growth of the frozen layer. Melt and mold temperatures influence the viscosity and the growth of the frozen layer, and thus the thickness over which the orientation is locked in. Gate position and part geometry determine whether the flow is locally diverging, converging, or shear-dominated, and they create regions such as weld lines in which the orientation deviates strongly from the global flow direction. Fiber content and aspect ratio affect the interaction intensity and hence both the steady-state alignment and the orientation kinetics [4,5,52,56].

These effects are coupled, and their individual contributions are difficult to separate experimentally. This is one of the main reasons why orientation models require material-specific calibration and why a Digital Twin cannot rely on empirical process–orientation correlations transferred from one part geometry to another.

## 4. Experimental Characterization of Fiber Architecture

**Figure 3:**
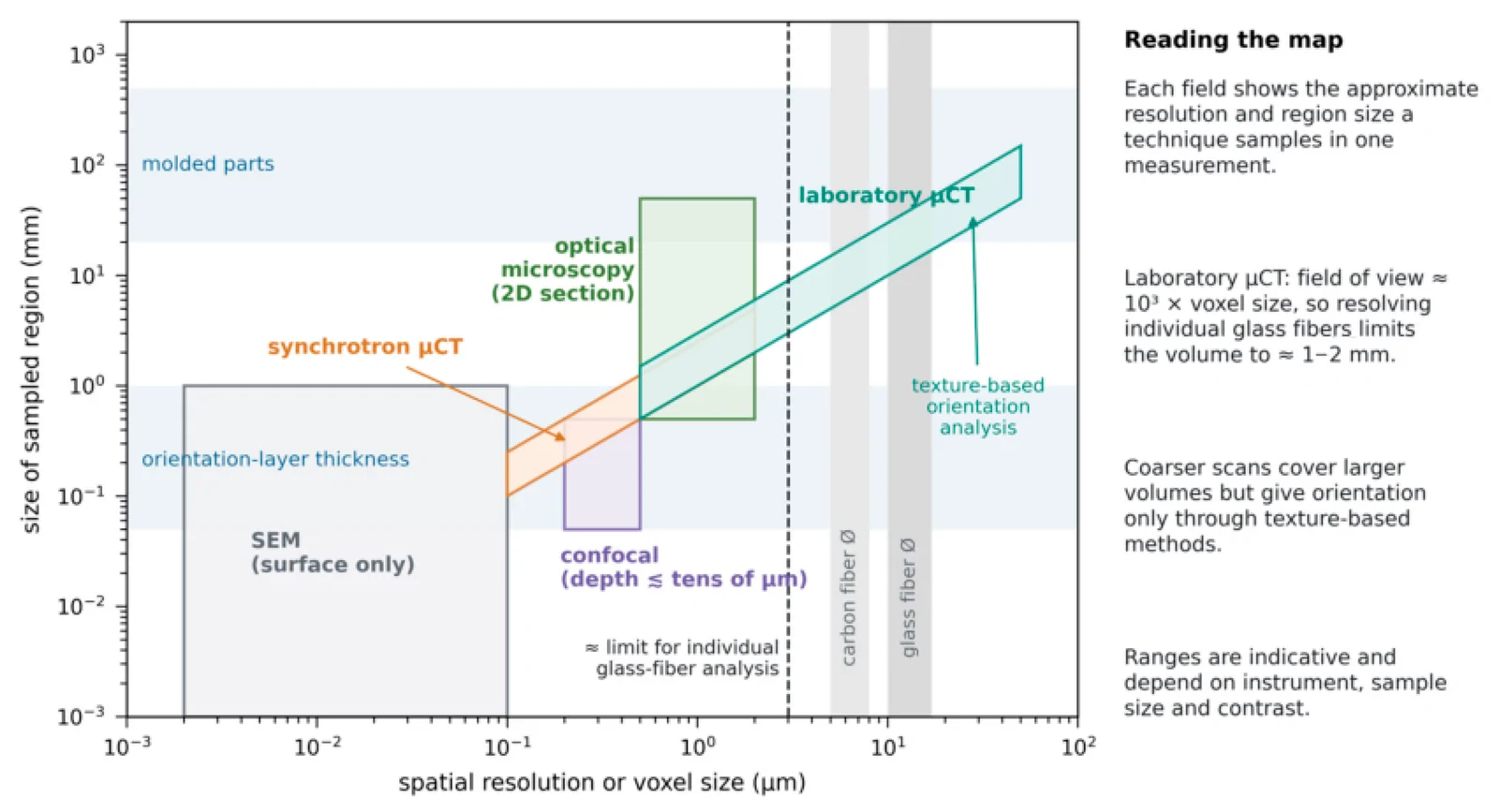
Approximate resolution and sampled region of experimental techniques for fiber-architecture characterization (schematic; logarithmic axes). Vertical bands indicate typical diameters of carbon and glass fibers; horizontal bands indicate the typical thickness of the orientation layers and the size of molded parts. Individual glass fibers can be resolved by laboratory µCT only in millimeter-sized volumes, whereas texture-based analysis of coarser scans extends orientation measurements to larger volumes. Ranges are indicative and depend on the instrument and sample.

In a Digital Twin context, characterization has two roles. It provides the reference against which process and orientation models are calibrated and validated, and it provides the only direct access to descriptors that are not predicted, such as local fiber content, curvature, and voids. The available methods differ in dimensionality, resolution, sampled volume, and throughput (Figure 3 and Table 5), and no single method satisfies all requirements.

### 4.1. Two-Dimensional and Near-Surface Methods

The classical method for measuring fiber orientation is optical microscopy of polished cross-sections. A fiber cut by the section plane appears as an ellipse whose axis ratio and inclination give the out-of-plane and in-plane angles of the fiber; averaging over many ellipses with appropriate weighting yields the orientation tensor [4,5]. The method is fast and inexpensive, but it is destructive, it cannot distinguish the sign of the out-of-plane angle, and its accuracy decreases for fibers nearly perpendicular to the section, which requires statistical corrections [39,57]. Confocal laser scanning microscopy removes the sign ambiguity by imaging the fiber path below the surface over a depth of some tens of micrometers, and it enabled some of the first automated, high-angular-resolution orientation measurements [58]. Scanning electron microscopy resolves fracture surfaces and interfaces at the nanometer scale and is indispensable for identifying failure mechanisms such as debonding and fiber pull-out, but it provides no volumetric architecture data [37].

Sharma et al. quantified the measurement uncertainty of the method of ellipses and propagated it through a Mori–Tanaka model, showing how errors in orientation measurement translate into uncertainty in predicted stiffness [57]. Such analyses are rare, but they provide exactly what a Digital Twin needs: an estimate of the confidence attached to each measured state variable.

### 4.2. X-Ray Computed Tomography

X-ray computed tomography reconstructs the three-dimensional attenuation field of a sample from a series of projections [25,59]. For glass fiber-reinforced polymers, the contrast between fiber and matrix is high, and laboratory µCT systems resolve individual fibers when the voxel size is small compared with the fiber diameter; voxel sizes of about 1 µm are typical for individual-fiber analysis of glass-fiber compounds [14,15]. Carbon fibers are more difficult to analyze because of their smaller diameter and lower contrast with the matrix. Synchrotron tomography offers higher flux, sub-micrometer resolution, and acquisition times short enough for in situ experiments that follow damage evolution under load [25,60], but access is limited and the samples are small.

The central limitation of µCT is the trade-off between resolution and field of view. A reconstructed volume typically contains on the order of 10^3^ voxels along each axis, so that a voxel size of 1 µm limits the field of view to roughly 1–2 mm [14,25]. This is small compared with the size of a molded part and comparable with the thickness of the orientation layers (Figure 3). Measurements therefore sample small regions of interest, and the statistical representativeness of these regions must be established explicitly. Lower-resolution scans of larger volumes can still provide orientation information through texture-based methods (Section 4.3), which do not require individual fibers to be resolved [61,62].

The resolution–volume trade-off directly affects the representativeness of the region of interest (ROI). Because the orientation varies through the thickness and in the plane, a single ROI is representative only of the position from which it was taken. It should span the full thickness of the part, or at least the layer of interest, and be large enough in the plane for the orientation tensor to be statistically converged. The required size can be estimated from the variance of the descriptor over subvolumes of increasing size, in analogy with the statistical definition of the representative volume element [63]. For second-order orientation tensors, this size is usually smaller than the scale of the variations caused by the part geometry, whereas length distributions and local fiber content require larger volumes, because long fibers are truncated at the ROI boundary. Representativeness for the whole component must therefore be achieved by sampling: several ROIs at locations with different flow kinematics—near the gate, in regions of nearly simple shear, at weld lines, ribs, and thickness changes—scanned and analyzed with the same protocol [40,54]. Multi-resolution strategies combine a coarse scan of a large volume with high-resolution scans of selected ROIs; Baranowski et al. obtained consistent local orientations from region-of-interest tomography at voxel sizes from 45 µm down to 3 µm, which extended the analyzable field of view by about an order of magnitude compared with conventional high-resolution scans [64]. The number of ROIs is ultimately an economic decision, which is one reason why model-based interpolation between sampled locations is required (Section 8.6).

**Table 5:** Experimental techniques for characterizing the fiber architecture of SFRPCs. Resolution and sampled-region values are indicative.

Technique	Information Obtained	Typical Resolution	Sampled Region	Main Limitations
Optical microscopy (method of ellipses)	2D section; orientation tensor, fiber content	≈0.5–1 µm	Section, mm^2^–cm^2^	Destructive; sign ambiguity; errors for fibers normal to the section [4,57]
Confocal laser scanning microscopy	Near-surface 3D fiber paths	≈0.2–0.5 µm (lateral)	Depth of tens of µm	Limited penetration depth [58]
Scanning electron microscopy	Fracture surfaces, interfaces	nm range	Surface only	No volumetric data [37]
Laboratory µCT	3D orientation, length, curvature, voids	≈0.5–5 µm voxel (≈1 µm for individual glass fibers)	≈1–2 mm field of view at 1 µm voxel; larger at coarser voxel	Resolution–volume trade-off; analysis effort; low contrast of carbon fibers [14,15]
Synchrotron µCT	As laboratory µCT; time-resolved and in situ	Sub-µm	Small volumes	Limited access; small samples [25,60]

### 4.3. From Reconstructed Volumes to Descriptors

Converting a reconstructed volume into model-ready descriptors is itself a modeling step with its own errors. Two families of methods are used. Texture-based methods compute a local structure tensor from the image gradients and derive a local orientation that is then averaged into orientation tensors; they are fast and robust and also work when fibers are not individually resolved [61]. Individual-fiber methods segment the fibers, separate touching fibers, and trace their center lines, which additionally yields fiber length, curvature, and position [15,65]. Hessman et al., for example, developed an iterative single-fiber segmentation and merging procedure for injection-molded polyamide 6.6 with 35 wt% glass fibers scanned at a voxel size of 1 µm, and validated the resulting fiber-length distributions against artificial data and incineration measurements [15].

Different algorithms applied to the same data do not yield identical orientation tensors. Pinter et al. compared several analysis methods on CT data of fiber-reinforced composites and estimated their errors [62], and Bernasconi et al. compared optical and tomographic methods on the same material [39]. The practical consequence is that the analysis method, its parameters, and the averaging volume should be reported with every measurement used for model calibration, and that measurement uncertainty should be propagated to the model parameters rather than ignored.

### 4.4. Implications for Digital Twins

Two conclusions follow for Digital Twin development. First, µCT should be regarded as a calibration and validation instrument rather than as an inline sensor: acquisition and analysis times of minutes to hours per region of interest are incompatible with production cycle times, so a Digital Twin will rely on µCT data from sampled parts and on models to interpolate between them. Second, the information that reaches the model is limited by the weakest step of the analysis—segmentation, separation of touching fibers, or spatial averaging—rather than by the nominal scanner resolution. Automating and validating this step is where machine learning has so far had its clearest impact (Section 7.1).

## 5. Numerical Modeling of Fiber Orientation

### 5.1. Description of the Orientation State

The orientation of a single rigid fiber is described by a unit vector **p** along its axis, and the orientation state of a fiber population by a probability distribution function *ψ*(**p**). Because solving for *ψ* at every point of a mold-filling simulation is too expensive, practical simulations use its moments, the orientation tensors [10]:

(1) *a_ij_* = ∮ *p_i_ p_j_ ψ*(**p**) d**p**,  *a_ijkl_* = ∮ *p_i_ p_j_ p_k_ p_l_ ψ*(**p**) d**p**

The second-order tensor **a** is symmetric and has unit trace; its eigenvectors give the principal orientation directions and its eigenvalues the degree of alignment. The component *a*_11_ in the flow direction is the quantity most often reported in experiments and simulations. The evolution equation for **a** contains the fourth-order tensor 𝔸, which must be approximated in terms of **a** by a closure approximation. The quadratic closure is exact only for perfect alignment and generally overestimates alignment; the hybrid closure [66], orthotropic fitted closures [67], the invariant-based optimal fitting (IBOF) closure [68], and the fast exact closure [69] provide progressively better accuracy. The closure is a source of error independent of the orientation model itself, and it should be reported together with the model parameters.

The following notation is used throughout this review. Scalars are written in italics (e.g., *C*_I_, *ξ*), vectors and second-order tensors in bold upright letters (e.g., **p**, **a**, **D**, **W**, **C**), and fourth-order tensors in blackboard bold (e.g., 𝔸). A single dot denotes a single contraction and a colon a double contraction, e.g., (𝔸:**D**)_ij_ = *a*_ijkl_ *D*_kl_, with summation over repeated indices. The rate-of-deformation and vorticity tensors are the symmetric and antisymmetric parts of the velocity gradient, *D*_ij_ = ½(∂*u*_i_/∂*x*_j_ + ∂*u*_j_/∂*x*_i_) and *W*_ij_ = ½(∂*u*_i_/∂*x*_j_ − ∂*u*_j_/∂*x*_i_), where **u** is the velocity field, and D/D*t* denotes the material time derivative.

### 5.2. Jeffery and Folgar–Tucker Models

Jeffery’s analysis of a rigid ellipsoid in a Newtonian fluid at vanishing Reynolds number gives the rotation rate of an isolated fiber [46]:

(2) $$\dot{p}=W\cdot p+\xi \left[D\cdot p-\left(p\cdot D\cdot p\right)p\right],\;\xi =\frac{r_{e}^{2}-1}{r_{e}^{2}+1}$$

where **D** and **W** are the rate-of-deformation and vorticity tensors and *r*_e_ is the effective aspect ratio; for slender fibers, *ξ* ≈ 1. Taking moments of Equation (2) and adding an isotropic rotary diffusion term that represents fiber–fiber interactions yields the Folgar–Tucker equation [10,47]:

(3) $$\frac{Da}{\mathrm{Dt}}=W\cdot a-a\cdot W+\xi \left(D\cdot a+a\cdot D-2A:D\right)+2C_{I}\dot{\gamma }\left(I-3a\right)$$

where $\dot{\gamma }={\left(2D:D\right)}^{1/2}$ is the scalar strain rate, **I** is the identity tensor, and *C*_I_ is the dimensionless interaction coefficient. *C*_I_ is fitted to experimental data; values of the order of 10^−3^–10^−2^ are typical for injection-molding compounds, and empirical correlations with the product of fiber volume fraction and aspect ratio have been proposed [5,48]. The Folgar–Tucker model reproduces the qualitative shell–core structure. With a single isotropic diffusion coefficient, however, it cannot simultaneously match the measured degree of alignment and the measured rate at which it develops: in practice, it predicts an orientation that evolves too quickly with strain and underestimates the width of the core layer [12,49,70].

### 5.3. Slow Orientation Kinetics and Anisotropic Rotary Diffusion

Two modifications address these shortcomings. The first slows down the orientation kinetics. In the reduced-strain closure (RSC) model, Wang et al. scaled the growth rates of the eigenvalues of **a** by a factor *κ* < 1 while leaving the rotation of its eigenvectors unchanged; unlike an earlier approach that simply scaled the entire right-hand side of the evolution equation, this formulation is objective [49]. Tseng et al. later proposed the retarding principal rate (RPR) term, which subtracts a fraction *α* of the eigenvalue rates in the principal frame of **a** and achieves a similar retardation [70].

The second modification replaces isotropic diffusion with an anisotropic rotary diffusion tensor **C**. In the general anisotropic rotary diffusion (ARD) form, the diffusion term of Equation (3) is replaced by [48]:

(4) $${\left(\frac{Da}{\mathrm{Dt}}\right)}_{\mathrm{ARD}}=\dot{\gamma }\left[2C-2\mathrm{tr}\left(C\right)a-5\left(C\cdot a+a\cdot C\right)+10A:C\right]$$

Phelps and Tucker expressed **C** as a polynomial in **a** and **D** with five coefficients and combined it with RSC-type retardation (ARD-RSC). The model described orientation in both short- and long-fiber thermoplastics better than the Folgar–Tucker model, but its parameters are difficult to identify uniquely [48]. Tseng et al. proposed an improved ARD (iARD) model with only two parameters—the fiber–fiber interaction coefficient *C*_I_ and a fiber–matrix interaction coefficient *C*_M_—and combined it with the RPR term (iARD-RPR) [70]. Because the original iARD tensor depended on the velocity gradient in a way that violates objectivity, a revised formulation based on the square of the rate-of-deformation tensor was subsequently introduced [71]. Favaloro and Tucker analyzed the family of ARD models systematically [72]. Several of these models are now available in commercial injection-molding software, and comparisons with µCT data show improved predictions of the core width and of orientation in complex geometries, provided that the parameters are calibrated for the material [52,54,56].

### 5.4. Flow–Fiber Coupling and Orientation-Dependent Viscosity

Most injection-molding simulations are decoupled: the velocity field is computed with an isotropic, generalized Newtonian viscosity, and the orientation is computed afterward from this field. In reality, aligned fibers make the suspension anisotropic, which modifies the velocity field and, in turn, the orientation, particularly near the flow front and in the core. Fully anisotropic constitutive equations are computationally expensive and numerically demanding. The informed isotropic (IISO) viscosity model of Favaloro et al. retains a scalar viscosity but makes it depend on the local orientation state and flow type, so that the dominant coupling effect—an increased resistance to extension along the fiber direction—can be captured at moderate cost [53,73]. IISO is a constitutive (viscosity) model rather than an orientation evolution equation, and it is used in combination with an orientation model such as iARD-RPR. Coupled simulations of this type predict a wider core and flow-front shapes that are closer to experimental observations than those of decoupled simulations [53,73].

Three levels of flow–fiber coupling should be distinguished, because they lead to different orientation fields near the flow front and in complex geometries. In a decoupled (one-way) formulation, the flow is solved with an orientation-independent viscosity, and the orientation is computed afterward along the resulting kinematics; the orientation cannot influence the velocity field, the shape of the flow front, or the extent of the core. In a sequentially (weakly) coupled formulation, flow and orientation are solved in a staggered manner within each time step or iteration: the orientation from the previous step updates an orientation-dependent viscosity or fiber-stress contribution, which is then used to compute the next velocity field. Orientation-dependent scalar viscosities such as IISO are typically used in this way and capture the dominant effect—the increased extensional resistance along the fibers—at a cost close to that of a decoupled simulation [53,73]. In a fully (strongly) coupled formulation, the anisotropic fiber stress enters the momentum equation and is solved simultaneously with the orientation, for example, with anisotropic viscosity tensors derived from mean-field descriptions of the suspension [74]; this is the most consistent but also the most expensive and numerically demanding option. The differences between the three levels are largest where the flow is extensional or changes direction rapidly—at the advancing front, near the gate, at ribs, and around inserts—because there the fiber-induced anisotropy modifies the kinematics that in turn determine the orientation; in nearly simple shear flows of thin walls, decoupled simulations remain an acceptable approximation [12,53]. For a Digital Twin, a decoupled model may therefore suffice for thin-walled parts, whereas parts with thick sections or strongly three-dimensional flow require at least sequential coupling, and the coupling level should be stated with every prediction.

### 5.5. Coupling with Fiber Length

The orientation models above assume a single aspect ratio, whereas real parts contain a length distribution that evolves through attrition (Section 2.3). Since the aspect ratio affects both *ξ* and the interaction coefficients, a consistent description requires orientation and length to be computed together. The attrition model of Phelps et al. provides a length-distribution field that can be combined with orientation predictions and passed to micromechanical models [34]. Validation of such coupled predictions is still limited, largely because simultaneous, spatially resolved measurements of orientation and length are scarce.

### 5.6. Comparative Assessment

Table 6 compares the main model families. Three observations are relevant for Digital Twins. First, the accuracy gained with more elaborate models is realized only after calibration, and the calibration data—usually µCT orientation profiles at a few locations—are themselves uncertain. Automated calibration workflows that couple the solver to an optimizer through a metamodel make this step reproducible; Rienesl et al., for example, determined the parameters of iARD-RPR and of an IISO-based formulation against CT orientation data from three regions of a Venturi-shaped part [19]. Second, numerical choices other than the orientation model matter: for a short glass fiber-reinforced polyamide, Oubellaouch et al. showed that two-dimensional meshes failed to capture the orientation in regions of non-simple shear flow, where three-dimensional meshes were required, and that the choice of parameter set changed the prediction error substantially [56]. Similarly, Żurawik et al. compared µCT measurements (voxel size 2.64 µm) of injection-molded polyamide 6 with 30 wt% short glass fibers with predictions of the Moldflow rotational diffusion (MRD) model and found distinct orientation regions in the shell and core that could not be reproduced by varying a single model parameter [55]. Third, all these models remain phenomenological; none of them predicts fiber clustering, local variations in fiber content, or curvature, so these descriptors must be supplied by measurements when they are needed.

Uncertainties of the orientation-model parameters are rarely reported in the studies compared here, which is why the comparison in Table 6 is qualitative and gives no confidence intervals. There are three reasons. First, most studies calibrate the parameters by deterministic optimization—manual tuning or metamodel-based minimization of the deviation from measured profiles—which returns a point estimate but no distribution [19,54]. Second, the calibration data are few and correlated: orientation profiles from a small number of µCT regions do not constrain all parameters independently, so that, for example, the interaction coefficient and the slow-kinetics parameter can compensate for each other and are not uniquely identifiable [48,56]. Third, the measured profiles themselves carry analysis-dependent errors that are seldom quantified (Section 4.3). The available evidence nevertheless indicates that parameter uncertainty is substantial: Oubellaouch et al. found that the prediction error of the RSC model changed from about 5% with default parameters to 26% with a literature parameter set [56], and Quintana et al. reported that the calibrated models did not reproduce the orientation in regions of variable thickness [54]. Bayesian calibration, which treats the parameters and the model discrepancy as random variables [75], would provide the missing uncertainty; it has already been used to infer the fiber orientation and matrix properties of SFRPCs from as few as three tensile tests [76]. Reporting parameter uncertainties should become standard practice, because they are an input to the uncertainty propagation discussed in Section 6.3.

## 6. Multiscale Modeling and Process–Structure–Property Integration

### 6.1. From Orientation Tensors to Local Stiffness

The standard approach to computing the local stiffness of an SFRPC from orientation data is a two-step homogenization [10,11]. First, the stiffness tensor **C**^UD^ of a hypothetical composite with the same fiber content and all fibers aligned is computed with a mean-field model. The Mori–Tanaka scheme [77,78] and the Tandon–Weng equations [79] are the most widely used and give good estimates of the aligned stiffness of typical SFRPCs [11]. Second, **C**^UD^ is averaged over the orientation distribution. For a transversely isotropic **C**^UD^, the orientation average depends only on **a** and 𝔸 [10]:

(5) *C_ijkl_* = *B*_1_*a_ijkl_* + *B*_2_(*a_ij_δ_kl_* + *a_kl_δ_ij_*) + *B*_3_(*a_ik_δ_jl_* + *a_il_δ_jk_* + *a_jl_δ_ik_* + *a_jk_δ_il_*) +*B*_4_*δ_ij_δ_kl_* + *B*_5_(*δ_ik_δ_jl_* + *δ_il_δ_jk_*)

where the scalars *B*_1_–*B*_5_ are functions of the components of **C**^UD^ and *δ*_ij_ is the Kronecker delta. Equation (5) is the practical link between the orientation tensors delivered by process simulation and the anisotropic elastic properties required by structural analysis. Because it requires 𝔸, the closure approximation also affects the predicted stiffness. Müller and Böhlke showed that effective stiffnesses estimated from second-order orientation tensors alone can deviate by up to 20% from those that also use fourth-order information, although the deviation was about 4% for experimentally measured microstructures [80].

The influence of the closure approximation on the predicted stiffness can be stated directly. Equation (5) is linear in 𝔸, so that any closure error in 𝔸 is transferred to the stiffness with the weight *B*_1_, which is the largest of the five coefficients for stiff, slender fibers. At a given second-order tensor, the quadratic closure always underestimates the dominant component *a*_1111_ (by Jensen’s inequality, *a*_1111_ ≥ *a*_11_^2^) and is exact only for perfect alignment; for the partially aligned states of shell and core layers, it therefore underpredicts the stiffness in the principal directions. Dray et al. compared several closures for computing the thermoelastic properties of an injection-molded short-fiber composite and found that orthotropic fitted closures reproduced the results obtained with the measured fourth-order tensor closely, whereas the linear, quadratic, and hybrid closures produced significantly larger deviations [81]. In the illustrative calculation of Section 6.3, the quadratic closure lowers the in-plane moduli by about 13–16% in a shell layer and by 22–24% in a core layer relative to a maximum-entropy reference with the same **a**—considerably more than the effect of typical measurement uncertainties of **a**. For stiffness predictions, orthotropic fitted, IBOF, or exact closures should therefore be preferred, and when µCT data are available, the fourth-order tensor should be computed directly from the measured fiber directions rather than closed.

For nonlinear behavior—plasticity, damage, and fatigue—mean-field models are extended with incremental formulations [82]. Their accuracy for strength and fatigue is, however, considerably lower than for stiffness, because failure depends on local features such as fiber ends, clusters, voids, and weak interfaces that orientation tensors do not describe [8,9]. Full-field simulations of representative volume elements (RVEs) generated from measured or synthetic microstructures resolve these features, but the RVE size required for statistical representativeness can be large [63], and the cost of full-field simulations prevents their use at every integration point of a component model.

The choice of the mean-field scheme matters less for elastic stiffness than the closure, but it becomes important at higher fiber contents and for nonlinear behavior (Table 7). Tucker and Liang found that the Mori–Tanaka (MT) model and the Lielens interpolation gave the best stiffness predictions for aligned short-fiber composites and that the MT estimate coincides with the lower Hashin–Shtrikman–Walpole bound [11]. The Lielens model interpolates between the MT estimate and its inverse, in which the roles of matrix and fibers are exchanged, with a weight that increases with the fiber volume fraction; it was introduced to improve predictions at higher fiber contents [83]. For misaligned fibers, the one-step MT scheme can produce non-symmetric effective stiffness tensors, which is why the two-step procedure of Equation (5) or the interpolative double-inclusion formulation is preferred [84]. For elastoplastic and viscoplastic matrices, incremental MT formulations linearize the matrix behavior at each load increment with tangent or secant operators; formulations for distributed-orientation fibers and second-moment formulations improve the predictions for strongly nonlinear matrices [82,85], and these models are the basis of the nonlinear mean-field analyses of injection-molded parts available in commercial multiscale software. Full-field computations on representative volume elements are the standard reference for assessing such schemes [86]; Karl and Böhlke found the two-step MT and the lower Hashin–Shtrikman estimates well suited for fiber volume fractions up to about 20% [74]. In the illustrative example of Section 6.3, the MT and Lielens estimates of the shell-layer moduli differ by less than 2%, whereas the inverse MT estimate is 41–84% higher, which shows that the scheme is a smaller source of uncertainty than the closure or the orientation state as long as the fiber content is moderate and an established scheme is used.

### 6.2. Mapping and Integrative Simulation

In an integrative simulation, the orientation field computed on the mold-filling mesh is mapped onto the structural mesh, and a local material model is assigned to each integration point [12,13]. Mapping between meshes of different type and resolution requires care, because component-wise interpolation of orientation tensors does not in general preserve their properties; dedicated interpolation schemes have been proposed for this purpose [87]. Görthofer et al. demonstrated a complete virtual process chain for sheet molding compound, in which the compression-molding simulation, orientation prediction, mapping, and mean-field-based structural simulation were validated step by step against µCT data and mechanical tests [13]. Validated chains of this kind form the computational core of any SFRPC Digital Twin.

### 6.3. Uncertainty Propagation

Every link of the chain introduces uncertainty: in the process boundary conditions, in the orientation model and its parameters, in the closure approximation, in the measured microstructure used for calibration, and in the homogenization model. Few studies propagate these uncertainties to the component level. Sharma et al. showed how orientation-measurement uncertainty propagates to predicted stiffness [57], and data-driven frameworks for the analysis of materials under uncertainty have been proposed more generally [88]. For a Digital Twin whose predictions are used to accept or reject parts or to change process settings, a quantified uncertainty is not optional; it is part of the prediction [24].

Table 8 summarizes the main sources of uncertainty along the chain, together with representative magnitudes reported in the literature or estimated in this review. The sources are not independent. The orientation-model parameters are calibrated against µCT data, so that the measurement error of the µCT descriptors enters the parameters; the closure approximation is used both in the orientation model and in the homogenization; and a systematic error of the mold-filling simulation, such as a wrong flow-front shape, shifts the orientation field in a spatially correlated way. Treating the sources as separate and adding their variances therefore misrepresents the total uncertainty. For a property *P* = *f*(*θ*_1_, …, *θ*_n_) predicted by the chain, such as a local modulus, a first-order (linearized) estimate of the variance makes these interactions explicit:

(6) $$\mathrm{Var}\left(P\right)\approx \sum_{i}{\left(\frac{\partial f}{\partial \theta_{i}}\right)}^{2}\sigma_{i}^{2}+2\sum_{i<j}\frac{\partial f}{\partial \theta_{i}}\frac{\partial f}{\partial \theta_{j}}\rho_{\mathrm{ij}}\sigma_{i}\sigma_{j}$$

where *θ*_i_ are the uncertain inputs (measured orientation, model parameters, fiber length and content, constituent properties), *σ*_i_ their standard deviations, and *ρ*_ij_ their correlation coefficients. The correlation terms express the interaction that is lost when the sources are treated separately: a µCT analysis that systematically overestimates *a*_11_, for example, leads to calibrated parameters that reproduce this bias, so that measurement and calibration errors are positively correlated and add up rather than averaging out. Model-form errors—closure, homogenization scheme, mesh, and coupling level—are not random variables in this sense; they are better represented by explicit discrepancy terms or by comparing alternative models, as in the Bayesian calibration framework of Kennedy and O’Hagan [75].

At the component level, uncertainties can be propagated in three ways. Sampling methods (Monte Carlo or Latin hypercube sampling) propagate the full input distributions through the chain and converge to the exact result for many samples, but they require surrogates when each evaluation involves a mold-filling simulation [88]; multilevel polynomial surrogates have recently been used to propagate uncertain material properties through injection-molding simulations to the fiber orientation with rigorous error bounds and at a fraction of the cost of standard Monte Carlo sampling [89]. Linearized propagation according to Equation (6) is inexpensive and indicates which sources dominate, but it fails for strongly nonlinear responses such as strength. Hierarchical approaches propagate uncertainty link by link—from µCT measurement to orientation parameters, from orientation to local stiffness, and from local stiffness to the component response—and are natural for a Digital Twin in which each link is updated with different data [57,75]. In all cases, the spatial correlation of the orientation field must be represented: independent errors at different integration points partly average out in the component stiffness, whereas correlated errors, such as a systematically wrong core width, do not.

**Table 8:** Sources of uncertainty along the process–structure–property chain of SFRPCs and representative magnitudes.

Link	Source	Type	Representative Magnitude or Evidence	Reduction or Quantification
Process inputs	Machine settings, melt and mold temperatures, material batch (viscosity, fiber content)	Parametric, random	Rarely reported in orientation studies	Cycle-wise process data; batch records; inputs treated as random variables
µCT acquisition and segmentation	Voxel size, noise, separation of touching fibers	Measurement; random and systematic	Fiber detection rate of 93.7% relative to optical ground truth for U-Net segmentation of a ceramic-matrix composite [90]; length distributions more sensitive than orientation (Section 7.1)	Multi-reference validation; synthetic ground truth; round robins
Orientation analysis	Algorithm (structure tensor or individual fibers), averaging volume	Measurement; systematic	Algorithm-dependent differences on the same data [62]	Reporting of analysis parameters; method comparison
Optical method of ellipses	Section plane, sign ambiguity, fibers normal to the section	Measurement	Uncertainty quantified and propagated to stiffness [57]	Statistical corrections; uncertainty propagation
ROI representativeness	Sampled volume versus part-scale variations	Sampling	Location-dependent; not captured by a single ROI (Section 4.2)	Several ROIs; RVE-size analysis [63]
Orientation-model parameters	Interaction, slow-kinetics, and anisotropy parameters calibrated on few profiles	Parametric; correlated with measurement	Prediction error of the RSC model 5% or 26% depending on the parameter set [56]	Bayesian calibration [75,76]; identifiability analysis
Orientation-model form, coupling, and mesh	Kinetics, flow–fiber coupling, 2D or 3D mesh	Model form; systematic	2D meshes failed in regions of non-simple shear flow [56]; shell and core regions not reproduced by varying a single parameter [55]	Model comparison; discrepancy models
Closure approximation	Quadratic, hybrid, fitted, or exact closure	Model form; systematic	Up to 20% (theoretical) and about 4% (measured microstructures) between second- and fourth-order-based estimates [80]; 13–24% in Table 9	Fitted or exact closures; measured fourth-order tensor
Mapping between meshes	Interpolation of tensors between meshes	Numerical	Component-wise interpolation can violate tensor properties [87]	Dedicated interpolation schemes [87]
Homogenization scheme	MT, Lielens, two-step, incremental	Model form	Below 2% between MT and Lielens for stiffness at 16 vol% (Table 9); larger for nonlinear response [85]	Full-field reference computations [86]
Fiber length and interface	Length distribution, interfacial strength	Parametric	Coefficient of variation of about 3.5% in *E*_11_ for 20% scatter of the aspect ratio (Table 9); interface rarely quantified (Section 2.2)	µCT length data [15]; interface tests [37]

An illustrative example is given in Table 9 for a polyamide 6 reinforced with 30 wt% (about 16 vol%) short glass fibers. The aligned stiffness was computed with the Mori–Tanaka scheme using the Eshelby tensor of prolate spheroids (Tandon–Weng) for an aspect ratio of 25, and orientation averaging was performed with Equation (5) for representative shell and core orientation states. Model-form choices (closure and homogenization scheme) were evaluated deterministically, and three input uncertainties—orientation (standard deviations of 0.03 in *a*_11_ and 0.01 in *a*_33_), aspect ratio (coefficient of variation of 20%), and fiber volume fraction (standard deviation of 0.8 vol%)—were propagated by Monte Carlo sampling with 1500 samples each. Each input uncertainty alone produces a coefficient of variation of about 3–3.5% in the shell modulus *E*_11_, and all three together produce about 6%, with a 95% interval of about 7.5–9.4 GPa around a nominal value of 8.4 GPa. The model-form choices produce larger, systematic deviations: −13% for the quadratic closure in the shell, −24% in the core, and +41% for the inverse Mori–Tanaka estimate. The numbers depend on the assumed inputs and are not general, but they illustrate two points that agree with the literature [57,80,81]: model-form errors can exceed parametric uncertainties, and the closure and the orientation state, rather than the choice between established mean-field schemes, dominate the uncertainty of stiffness predictions.

**Table 9:** Illustrative propagation of model-form choices and input uncertainties to the predicted Young’s moduli (GPa) of a polyamide 6 with 30 wt% short glass fibers. Deviations from the reference are given in parentheses.

Case	Shell *E*_11_	Shell *E*_22_	Core *E*_11_	Core *E*_22_
Model-form choices (deterministic)				
Reference: MT + maximum-entropy fourth-order tensor	8.44	4.74	5.23	7.29
Quadratic closure	7.34 (−13.1%)	3.98 (−16.1%)	3.97 (−24.1%)	5.67 (−22.2%)
Hybrid closure	7.37 (−12.7%)	4.10 (−13.4%)	4.46 (−14.8%)	6.16 (−15.5%)
Linear closure	7.33 (−13.1%)	4.57 (−3.6%)	5.11 (−2.2%)	6.73 (−7.6%)
Lielens scheme instead of MT	8.56 (+1.4%)	4.82 (+1.7%)	5.32 (+1.7%)	7.40 (+1.5%)
Inverse MT instead of MT	11.89 (+40.9%)	8.72 (+84.2%)	9.23 (+76.4%)	11.03 (+51.3%)
Input uncertainties (Monte Carlo, N = 1500; shell E_11_: mean, coefficient of variation, 95% interval)				
Orientation (σ = 0.03 in *a*_11_, 0.01 in *a*_33_)	8.45; 3.3%; 7.94–9.00			
Aspect ratio (coefficient of variation 20%)	8.39; 3.5%; 7.74–8.87			
Fiber volume fraction (σ = 0.008)	8.45; 3.4%; 7.92–9.00			
All three combined	8.39; 5.9%; 7.47–9.40			

Assumptions: matrix *E* = 3.0 GPa, *ν* = 0.38 (polyamide 6, dry, indicative); E-glass *E* = 72 GPa, *ν* = 0.22; fiber volume fraction 0.16; aspect ratio 25. Shell **a** = diag(0.75, 0.20, 0.05) and core **a** = diag(0.30, 0.62, 0.08), with direction 1 = flow and 3 = thickness. Aligned stiffness from the Mori–Tanaka scheme with the Eshelby tensor of prolate spheroids (Tandon–Weng) [79]; orientation averaging with Equation (5) [10]; reference fourth-order tensor from the maximum-entropy distribution with the given **a** [80]; hybrid closure weight 1 − 27 det **a** [66]; Lielens weight (*v* + *v*^2^)/2 [83]. Calculated by the authors for illustration; not a validation against measurements.

### 6.4. Guidance for Selecting Characterization and Modeling Methods

**Figure 4:**
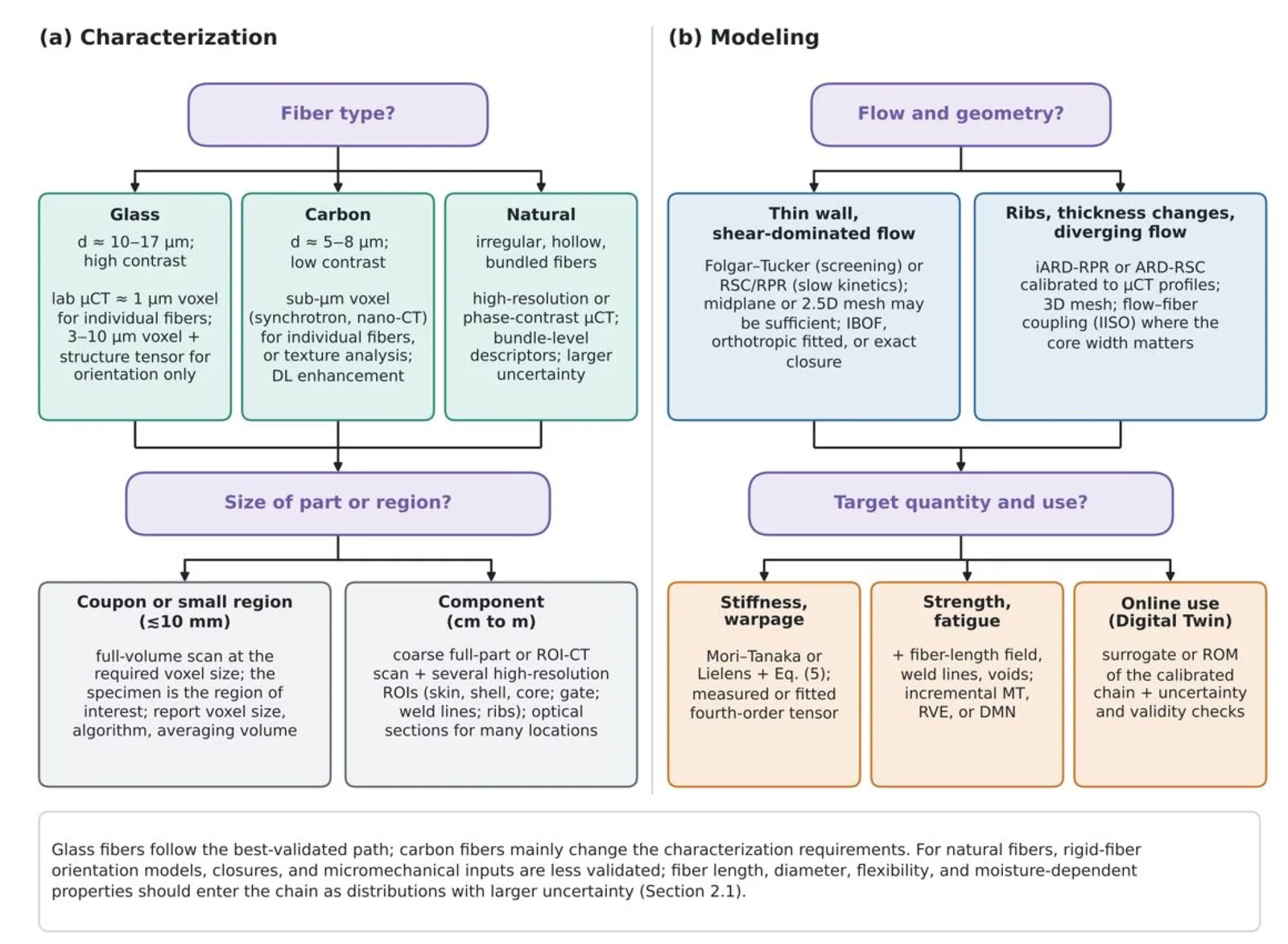
Decision tree for selecting (**a**) characterization and (**b**) modeling methods for SFRPC parts as a function of fiber type, part or region size, flow situation, and target quantity. The recommendations summarize Section 2, Section 3, Section 4, Section 5 and Section 6; resolution values are indicative. DL, deep learning; MT, Mori–Tanaka; ROI, region of interest; ROM, reduced-order model.

The choice of characterization and modeling methods depends mainly on the fiber type, the size of the part or region of interest, the flow situation, and the target quantity. Figure 4 condenses Section 2, Section 3, Section 4, Section 5 and Section 6 into a decision tree. On the characterization side (Figure 4a), the fiber type determines the resolution required: individual glass fibers can be resolved by laboratory µCT at a voxel size of about 1 µm, whereas carbon fibers require sub-micrometer voxels or texture-based analysis, and natural fibers require descriptors at the bundle level. The size of the part determines the sampling strategy: coupons can be scanned completely, whereas components require a multi-resolution strategy with several ROIs at locations of different flow kinematics (Section 4.2). On the modeling side (Figure 4b), thin-walled parts with predominantly shear flow can be described with the Folgar–Tucker or RSC model on midplane or 2.5D meshes, whereas parts with ribs, thickness changes, or diverging flow require calibrated anisotropic-diffusion models, three-dimensional meshes, and, where the core width matters, flow–fiber coupling (Section 5.3, Section 5.4, Section 5.5 and Section 5.6). The target quantity then determines the homogenization: two-step Mori–Tanaka or Lielens schemes with a measured or fitted fourth-order tensor for stiffness and warpage; additional length, defect, and nonlinear models for strength and fatigue; and surrogates or reduced-order models with uncertainty estimates for online use in a Digital Twin. The decision tree is a starting point rather than a prescription; borderline cases, such as moderately thick parts or high fiber contents, should be resolved by comparing alternative models against measurements.

## 7. Artificial Intelligence Along the Process–Structure–Property Chain

**Figure 5:**
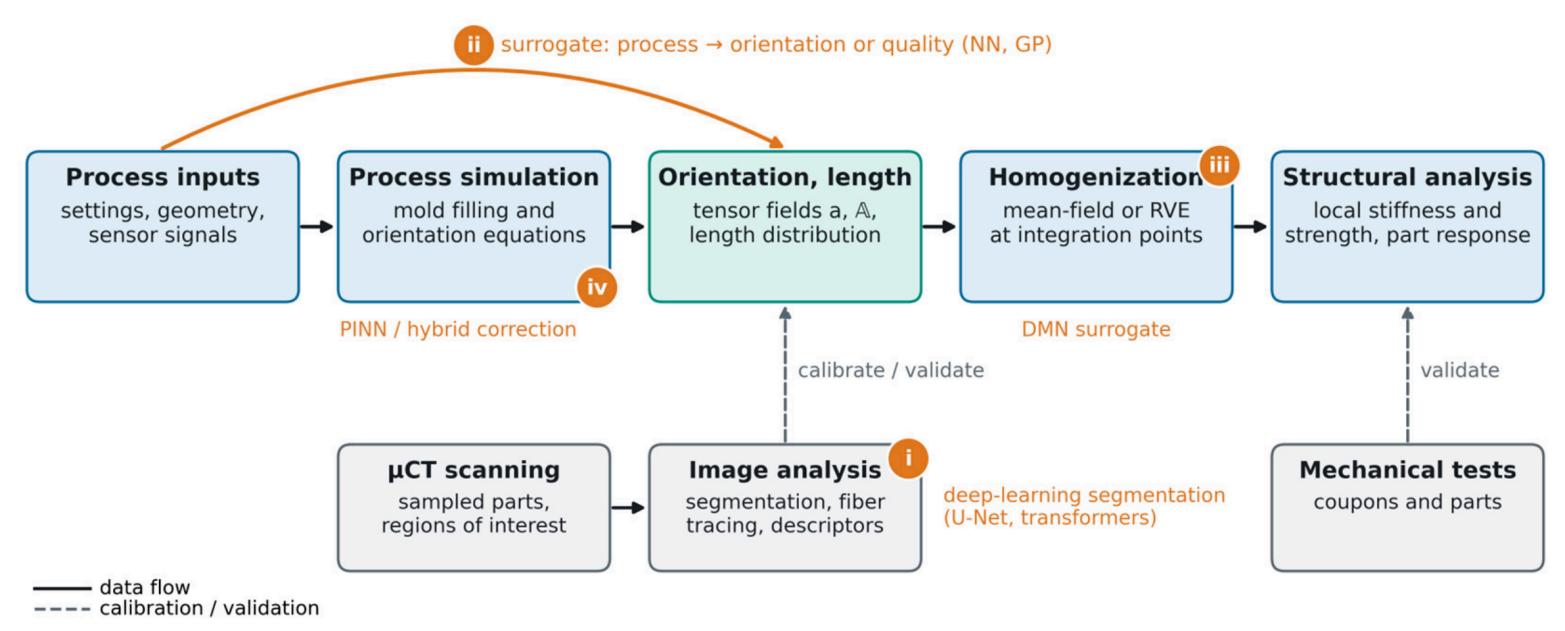
Entry points of machine learning in the experimental–computational workflow for SFRPCs: (i) image-based characterization, (ii) process–orientation and process–quality surrogates, (iii) micromechanical surrogates, and (iv) physics-informed and hybrid models. Solid arrows denote the main data flow; dashed arrows denote calibration and validation.

Machine learning enters the process–structure–property chain at four points (Figure 5): (i) the conversion of images into microstructural descriptors; (ii) the prediction of orientation or part quality from process parameters and geometry; (iii) the replacement of expensive micromechanical calculations by surrogates; and (iv) the combination of data with governing equations. Table 10 summarizes representative studies, and the following subsections discuss their contributions and limitations.

### 7.1. Image-Based Characterization

Segmentation of individual fibers in µCT volumes is the step in which deep learning offers the clearest advantage over conventional image processing, because fibers in SFRPCs touch and cross each other and have low contrast at practical resolutions. Encoder–decoder convolutional networks of the U-Net type [91] and, more recently, transformer-based architectures for volumetric data [92,93] are the main building blocks. Konopczyński et al. used three-dimensional deep embedding learning for the instance segmentation of fibers in low-resolution CT scans of short glass fiber-reinforced polymers [16]. Guo et al. combined unsupervised image enhancement based on a cycle-consistent generative adversarial network with a U-Net-based fiber identification network and obtained, from fast scans, a segmentation quality comparable to that of scans requiring a 200-fold longer acquisition time [17]—a result directly relevant to reducing the cost of µCT characterization. Ali et al. used deep-learning tools to convert µCT images of woven fabrics into digital material twins for simulation [94].

Validation remains the weak point. Badran et al. validated U-Net segmentation of CT images of ceramic-matrix composites against synthetic images, manual segmentation, and high-resolution optical images of the same sections, and obtained a fiber detection rate of 93.7% relative to the optical ground truth [90]. Comparable multi-reference validation is rarely reported for SFRPCs, where the output of interest is not a segmentation mask but the derived orientation and length distributions. Errors in the separation of touching fibers propagate directly into the length distribution, which is more sensitive to segmentation errors than the orientation tensor. Synthetic datasets with known ground truth can partly fill this gap: García-Gascón et al. published 55,092 synthetic two-dimensional images of fiber-reinforced microstructures annotated with their orientation tensors for training and benchmarking orientation-estimation models [95].

### 7.2. Process–Orientation and Process–Quality Surrogates

Neural networks can learn the mapping from process parameters and geometry to orientation fields or quality indicators and can thus replace repeated simulations. Herrmann et al. trained dense, convolutional, and long short-term memory (LSTM) networks on simulated injection-molding data to predict local orientation tensors and their dependence on geometry; the LSTM network performed best and reduced the computation time by about four orders of magnitude relative to the simulation [18]. Because the networks were trained exclusively on simulation results, their accuracy with respect to real parts is bounded by that of the underlying orientation model. This illustrates a general point: a surrogate reproduces its training data, not the physics.

At the process level, multilayer perceptrons trained on features extracted from process signals such as pressure curves predict part quality [96], and transfer learning can reduce the number of experiments needed when a model is moved to a new mold or material [97]. Surrogate-based and Bayesian optimization methods are established tools for process design [98,99,100], and their application to both discontinuous and continuous fiber composite processes has recently been reviewed [26]. The automated calibration of orientation models against µCT data is a related application, in which a metamodel of the simulation replaces the solver inside the optimization loop [19].

### 7.3. Micromechanical Surrogates

The homogenization step described in Section 6 must be evaluated at millions of integration points in a component model, which motivates surrogates that preserve the underlying mechanics. Deep material networks (DMNs) represent the RVE response by a hierarchical network of laminate building blocks with analytical homogenization; they are trained on linear elastic RVE data and then used to predict nonlinear behavior [20]. Gajek et al. analyzed the micromechanical foundations of DMNs [101] and developed an FE–DMN method for short fiber-reinforced components with spatially varying orientation, in which DMNs trained for representative orientation states replace full-field RVE simulations at the integration points [21]. Wei et al. implemented a DMN-based multiscale method for injection-molded SFRPCs in the commercial finite-element code LS-DYNA [102]. These methods are notable because physical structure is built into the network architecture, which improves extrapolation compared with generic regression models.

### 7.4. Physics-Informed and Hybrid Learning

Physics-informed neural networks (PINNs) include the residuals of the governing equations in the training loss and can therefore be trained with little or no labeled data [103,104]. In composite manufacturing, PINNs have been applied to the thermochemical curing of composite–tool systems [105] and as data-free surrogates for process optimization, where a PINN-based surrogate made optimization more than 500 times faster than with conventional simulation [106]. To the best of our knowledge and within the literature covered by the search protocol of Section 1.2 (last update October 2026), PINNs have not yet been applied to fiber-orientation evolution in injection molding. Related developments exist, however: Yun et al. learned macroscopic flow models of short-fiber suspensions from fine-scale simulation data [107], Ramoa et al. trained a physics-informed, data-driven closure relation for dilute fiber suspensions in homogeneous flows under admissibility constraints [108], and Zheng et al. used a physics-informed neural network to generate representative volume elements with non-uniform fiber distributions at high volume fractions [109]. None of these studies solves the coupled flow–orientation problem of mold filling. The orientation equations (Equations (3) and (4)) are first-order hyperbolic equations along streamlines, coupled to a free-surface flow, which makes them a challenging but well-defined target. A realistic intermediate step is hybrid modeling, in which a conventional solver provides the flow field and a learned correction accounts for systematic discrepancies between simulated and measured orientation.

A PINN formulation of the coupled problem can be outlined as follows. A network with parameters **θ** maps the space–time coordinates (**x**, *t*) to the velocity **u**, the pressure *p*, a volume-of-fluid or level-set function *φ* that tracks the melt front, and the orientation tensor **a**. To guarantee that **a** remains symmetric, positive semidefinite, and of unit trace, the network can output an orthogonal tensor **Q** and three unnormalized eigenvalues that are passed through a softmax function, so that **a** = **Q** diag(*λ*_1_, *λ*_2_, *λ*_3_) **Q**^T^ with *λ*_i_ ≥ 0 and *λ*_1_ + *λ*_2_ + *λ*_3_ = 1. For the creeping flow of a generalized Newtonian melt, the residuals of the governing equations are

(7) $$r_{c}=\nabla \cdot u,\;r_{m}=-\nabla p+\nabla \cdot \left[2\eta \left(\dot{\gamma },T,a\right)D\right],\;r_{\varphi }=\frac{\partial \varphi }{\partial t}+u\cdot \nabla \varphi ,\;r_{a}=\frac{Da}{\mathrm{Dt}}-\left[W\cdot a-a\cdot W+\xi \left(D\cdot a+a\cdot D-2A:D\right)+2C_{I}\dot{\gamma }\left(I-3a\right)\right]$$

where the orientation-dependent viscosity *η* represents flow–fiber coupling (e.g., IISO) and reduces to an orientation-independent viscosity in a decoupled formulation, 𝔸 is obtained from a closure, and the bracket can be replaced by the right-hand side of an RSC or ARD model. The loss function combines these residuals with boundary, initial, and measurement terms:

(8) $$L\left(\theta \right)=\lambda_{c}{∥r_{c}∥}^{2}+\lambda_{m}{∥r_{m}∥}^{2}+\lambda_{\varphi }{∥r_{\varphi }∥}^{2}+\lambda_{a}{∥r_{a}∥}^{2}\;+\lambda_{b}{∥B\left(u,\varphi ,a\right)∥}^{2}+\lambda_{d}\sum_{k}{∥a\left(x_{k}\right)-a_{k}^{\mathrm{µCT}}∥}^{2}$$

where the norms denote mean squares over collocation points, *λ* are weighting factors, ℬ collects the inlet, wall, front, and initial conditions (including the orientation at the gate), and the last term enforces agreement with orientation tensors **a**_k_^µCT^ measured by µCT at the locations **x**_k_. Unknown model parameters, such as *C*_I_ or the slow-kinetics factor, can be included in **θ** and identified together with the fields, which turns the PINN into an inverse solver for orientation-model calibration [103,104]. The energy equation and a temperature-dependent viscosity can be added in the same way.

Several technical barriers make a complete PINN of mold filling a long-term goal: the moving melt front and the frozen layer introduce sharp interfaces that PINNs resolve poorly; the orientation equation is hyperbolic and transported along streamlines, which is difficult to learn with residual-based training; the loss terms differ by orders of magnitude and require adaptive weighting; and training must be repeated for each geometry unless operator-learning architectures are used. Three hybrid physics–ML strategies are more realistic in the near term. The first are calibration hybrids, in which the conventional solver is kept and ML is used only as a metamodel for parameter identification or as a Bayesian emulator [19,76]; such approaches are already in use. The second are learned closures or correction terms inside a conventional solver—for example, a neural network that replaces the closure approximation or the diffusion tensor while respecting admissibility constraints [108]—which keeps the transport structure of the solver and limits the learning task to a local constitutive relation. The third are discrepancy models, in which a statistical model, such as a Gaussian process, learns the systematic difference between simulated and measured orientation as a function of position and process conditions [75]. Operator-learning networks such as DeepONet [110], trained on calibrated simulations to map process conditions and geometry to orientation fields, are a medium-term option, whereas a fully physics-informed solution of the coupled problem remains a long-term research goal.

### 7.5. Trustworthiness: Uncertainty, Domain of Validity, and Explainability

Three properties determine whether an ML model can be used inside a Digital Twin. The first is uncertainty: each prediction should carry a confidence estimate, which Gaussian-process surrogates provide naturally and neural networks provide only with additional measures such as ensembles or Bayesian formulations [88,99]. The second is the domain of validity: a model trained on one part family, material, or machine should detect when it is queried outside its training domain. This is particularly important for process–orientation surrogates, whose training data rarely cover the variety of geometries found in production. The third is explainability: post hoc methods such as SHAP [111], LIME [112], and Grad-CAM [113] indicate which inputs or image regions drive a prediction and can reveal models that respond to artifacts rather than to physically meaningful features. Because these methods approximate the model behavior and can disagree with one another, they support but do not replace physical validation.

Table 11 complements Table 10 with the quantitative information reported in the original studies—accuracy, speed, and the amount and type of training data—and with the range for which the results were demonstrated. Two observations follow. First, the reported accuracies cannot be compared directly across studies, because they refer to different outputs (segmentation masks, orientation components, temperatures, or stiffness) and to different metrics. Second, the training data range from a few annotated µCT volumes to hundreds of simulations, and in most studies the demonstrated validity range is a single material and a narrow family of geometries.

**Table 10:** Representative applications of artificial intelligence to the fiber architecture and processing of fiber-reinforced composites.

Task	Study	Input → Output	Method	Main Result	Main Limitation
Fiber instance segmentation	Konopczyński et al. [16]	Low-resolution CT of short glass fiber composite → individual fibers	3D deep embedding network	Individual fibers from low-resolution scans	Segmentation only; descriptors not validated; evaluated with segmentation metrics on one material and resolution (fiber diameter 10–14 µm, 3.9 µm voxels), not by the accuracy of derived orientation and length distributions
Image enhancement and segmentation	Guo et al. [17]	Fast CT scans → segmented fibers	CycleGAN + U-Net	Quality comparable to 200× longer scans	Not yet demonstrated for SFRPCs; unpaired fast and slow synchrotron scans of one carbon-fiber composite; transfer to laboratory µCT and glass-fiber SFRPCs untested; artifacts generated by the enhancement network must be excluded
Validation of segmentation	Badran et al. [90]	CT of ceramic-matrix composite → fiber mask	U-Net	93.7% detection vs. optical ground truth	Different material class; ceramic-matrix composite with coated fibers, with lower detection for thinner coatings; transfer to polymer matrices and to orientation descriptors not shown
Digital material twin	Ali et al. [94]	µCT of woven fabric → simulation model	Deep-learning segmentation	Automated model generation	Textile architecture; woven-fabric geometry (yarns rather than individual fibers); new training data needed for SFRPCs
Orientation surrogate	Herrmann et al. [18]	Geometry and process → orientation tensor	Dense, CNN, LSTM networks	≈10^4^-fold speed-up	Trained on simulations only; 200 Folgar–Tucker plate simulations with one circular hole; accuracy bounded by the simulation and not compared with measured orientation; validity limited to the sampled plate geometries
Orientation-model calibration	Rienesl et al. [19]	CT orientation data → model parameters	Metamodel + hybrid optimization	Reproducible calibration	Single geometry; one material and one Venturi-shaped part with three CT regions; parameter uncertainty and identifiability not reported; transfer of parameters to other geometries untested
Quality prediction	Ke and Huang [96]	Process signals → part quality	Multilayer perceptron	Inline quality estimate	No microstructural information; quality indices from cavity-pressure curves; global quality indicators (part widths) rather than local properties; transfer to other molds requires new data
Transfer learning	Lockner and Hopmann [97]	Data from a new process setting → quality model	Transfer-learning ANN	Fewer experiments needed	Transferability limits; benefit depends on the similarity between source and target processes; no microstructural outputs
Micromechanical surrogate	Gajek et al. [21]; Wei et al. [102]	Orientation state → nonlinear local response	Deep material network	Component-scale multiscale analysis	Trained on RVE data; one network per microstructure class requires offline RVE training; accuracy for failure and fatigue less established; orientation input inherits process-simulation errors
Physics-informed surrogate	Würth et al. [106]	Process parameters → process fields	PINN without labeled data	>500× faster optimization	Not yet applied to orientation; single process and parametrized geometry; retraining needed for new geometries; no moving fronts or hyperbolic transport

**Table 11:** Quantitative performance, training data, and demonstrated validity range of representative ML applications (values as reported in the original studies; metrics are not directly comparable).

Study	Task	Training Data (Type and Amount)	Reported Accuracy	Speed	Demonstrated Validity Range
Konopczyński et al. [16]	Fiber instance segmentation	Low-resolution CT volumes of a short glass fiber composite with reference fiber labels (about 6500 fibers per test volume)	Adjusted Rand index 0.90 vs. 0.35 for a watershed baseline	n.a.	One material; fiber diameter 10–14 µm; 3.9 µm voxels
Guo et al. [17]	Image enhancement and segmentation	Unpaired fast and slow synchrotron scans (CycleGAN); U-Net trained on enhanced data	Segmentation comparable to that of scans with 200× longer acquisition	Acquisition time reduced about 200-fold	Carbon-fiber composite; time-resolved synchrotron CT
Badran et al. [90]	Segmentation and its validation	CT images of a SiC/SiC composite; synthetic images and manual labels; optical ground truth	93.7% fiber detection relative to optical images	n.a.	One ceramic-matrix composite; detection decreases for thinner fiber coatings
Herrmann et al. [18]	Process and geometry → orientation	200 Folgar–Tucker simulations of plates (170/15/15 for training, validation, test)	Mean squared error 7 × 10^−4^ (training) and 2.5 × 10^−3^ (validation)	About 10^4^-fold (seconds instead of hours)	Plates 50 mm wide, 60–90 mm long, 2–3.5 mm thick, with one hole
Rienesl et al. [19]	Calibration of orientation-model parameters	CT orientation data from three regions of one part; metamodel of the simulation	Calibrated iARD-RPR and IISO-based parameters (point estimates)	Fewer solver calls than direct optimization	One material and part geometry
Ke and Huang [96]	Process signals → part quality	Quality indices extracted from cavity-pressure curves	Accuracy above 92% for part widths	Inline	Molded parts of the original study
Hürkamp et al. [114]	Process parameters → interface temperature	100 FE simulations (Latin hypercube sampling); random forest	n.a.	Real time	One overmolding process and part
Hürkamp et al. [115]	Process parameters → temperature fields	90 FE simulations; POD with decision trees	n.a.	Real time	Thermoforming of one part family
Fernández-León et al. [116]	Pressure signals → filling state (RTM)	Synthetic Darcy-flow simulations; encoder–decoder networks	Errors below 1% (synthetic test data)	Online	One mold with five pressure sensors
Thomas et al. [76]	Tensile tests → orientation and matrix modulus	Hierarchical Bayesian model with micromechanics; as few as three tensile tests	Posterior distributions (uncertainty quantified)	n.a.	Extrusion deposition additive manufacturing; elastic properties
Würth et al. [106]	Process parameters → process fields	No labeled data (physics residuals only)	n.a.	Optimization more than 500-fold faster	One thermochemical process and parametrized geometry
Gajek et al. [21]; Wei et al. [102]	Orientation state → nonlinear response	Linear-elastic RVE simulations for each orientation state	Close agreement with full-field RVE simulations	Component-scale two-scale analyses feasible	Short-fiber composites; orientation states covered by training

n.a., not available in a form comparable with the other entries.

### 7.6. Data Requirements, Transferability, and Simulation Bias

Table 11 shows that the data requirements differ by orders of magnitude between applications. Segmentation networks are trained on a few annotated µCT volumes; because each volume contains thousands of fibers, the effective sample size is large, but the diversity of materials and imaging conditions is small. Process–orientation surrogates require hundreds of simulations for a single family of plate geometries [18], and micromechanical surrogates require RVE simulations for each microstructure class [21]. Experimental datasets with paired process, microstructure, and property information are the scarcest. The Bayesian approach of Thomas et al., which infers orientation and matrix properties from as few as three tensile tests, shows how prior physical knowledge can compensate for small datasets [76].

Transferability must be assessed along four axes: new materials (fiber type, content, and length; matrix rheology), new geometries (wall thickness, ribs, gates, weld lines), new machines and molds (sensor positions, thermal behavior), and new processing conditions outside the training range. A model that transfers well along one axis may fail along another, and high internal accuracy is not evidence of transferability, particularly when training and test data are not independent. Data leakage—information from the test set entering the training, for example, through random splits of spatially correlated points from the same part, of time steps from the same simulation, or of images from the same specimen—is a common cause of overoptimistic accuracy in ML-based science [117]. For SFRPC applications, test data should therefore be separated by group—whole parts, geometries, molds, materials, or µCT specimens—rather than by individual points, analogous to the blocked cross-validation recommended for structured data [118], and the validity range should be reported along each of the four axes.

Surrogates trained on simulations inherit the bias of the simulations. Herrmann et al. noted that their networks cannot be better than the simulations used for training [18]. In a Digital Twin, such a surrogate reproduces the systematic errors of the orientation model—for example, an underestimated core width (Section 3.2)—with high confidence, because its own uncertainty estimate refers only to the deviation from the simulation, not from reality. Three measures mitigate this problem: training on simulations whose parameters have been calibrated against µCT data, which reduces the bias at the source; adding an explicit discrepancy model learned from measurements [75]; and multi-fidelity training, in which many simulations are combined with few measurements so that the measurements correct the simulation-based trend. Transfer learning, which adapts a pretrained model with a small number of new experiments [97], addresses changes in process or mold but not the bias of the simulation itself. Synthetic datasets with known ground truth, such as the annotated images of García-Gascón et al. [95], are valuable for benchmarking but carry the same risk if the synthetic generator does not represent real microstructures.

## 8. Digital Twins for SFRPC Manufacturing

### 8.1. Definitions and Levels of Integration

The term Digital Twin is used inconsistently. Grieves and Vickers described it as a virtual representation of a physical product that is linked to it by information exchange throughout its life cycle [22]. Kritzinger et al. distinguished three levels by the direction and automation of the data flow: a *digital model* exchanges no data automatically with the physical object; a *digital shadow* receives data automatically from the physical object, but changes in the model do not act back automatically; and a *digital twin* is coupled to the physical object automatically in both directions [23]. A report of the U.S. National Academies emphasized that a Digital Twin must also include verification, validation, and uncertainty quantification (VVUQ) and must be fit for a specific decision-making purpose [24]. Kalidindi et al. extended the concept to materials, defining a materials digital twin as a representation of the evolving multiscale structure and properties of a specific material instance [31]. For SFRPCs, both perspectives meet: a process twin (machine, mold, and process states) and a material twin (fiber architecture and local properties of each part). In this review, the terms *digital model*, *digital shadow*, and *digital twin* (lowercase) are used strictly in the sense of Kritzinger et al. [23], whereas the capitalized term Digital Twin refers to the concept in general; when an original work uses a different term, the term used by its authors is given in Table 12.

### 8.2. State of Implementation

Table 12 classifies representative implementations in composite and polymer manufacturing according to the scheme of Kritzinger et al. [23]. Most systems are digital models or digital shadows that combine an offline-trained surrogate of a physics-based simulation with process data to provide fast predictions and decision support. Hürkamp et al., for example, trained a random-forest surrogate on 100 finite-element simulations of the overmolding of thermoplastic composite inserts to predict interface temperatures—a proxy for bond quality—from production parameters [114], and used proper orthogonal decomposition combined with decision trees to provide temperature fields in real time for thermoforming [115]. Other systems link measured microstructural data to structural simulation: Seon et al. mapped CT-measured void distributions of prepreg layups into finite-element models to simulate and mitigate void formation during debulking [119], and Zambal et al. linked manufacturing data of composite parts to an effects-of-defects analysis [120]. Frameworks for injection molding that integrate sensors, machine learning, and case-based reasoning have also been proposed [121,122], but they address machine and process states rather than the internal fiber architecture.

The integration level in Table 12 was assigned with three explicit criteria derived from Kritzinger et al. [23]: (i) the direction of the data flow between the physical process and the model—none, physical-to-digital only, or both directions; (ii) whether each direction is automatic, that is, executed without manual data transfer or human intervention during operation; and (iii) whether the data refer to the specific physical instance (a particular machine, mold, or part) rather than to a process or material class. A system was classified as a *digital model* if no automatic data flow from the physical process was documented, including simulation chains validated offline; as a *digital shadow* if measured data of the specific instance were transferred automatically to the model but model outputs reached the process only through a human decision; and as a *digital twin* if, in addition, model outputs acted back on the process automatically, for example, through set-point changes. Where the published description did not allow a clear decision, the lower level was assigned, and systems without a reported implementation were marked as concepts. The column “Term used in original work” in Table 12 gives the designation chosen by the original authors, which often differs from this classification.

Further implementations from composite manufacturing and closely related fields extend this picture (Table 12). In liquid composite molding, Fernández-León et al. developed a digital twin of resin transfer molding (RTM) in which deep-learning encoder–decoder surrogates, trained on synthetic Darcy-flow data, monitor the filling online from five pressure sensors, with errors below 1% reported for synthetic test data [116], and Iglesias et al. formulated the online estimation of preform permeability during RTM as a Bayesian inverse problem solved with ensemble Kalman and sequential Monte Carlo methods [123]. In curing, Yokoyama et al. and Matsuzaki et al. used ensemble Kalman filtering to estimate the curing heat of composites from temperature measurements and to optimize localized heating [124,125]. In injection molding, self-optimizing machines that adapt the process from cavity-pressure measurements by iterative learning control close the loop on process states rather than on the microstructure [126]. In metal additive manufacturing—a related field in which the microstructure is also created by the process and cannot be observed directly—digital twins have been proposed that combine mechanistic process models with sensing, statistical, and data-driven components for rapid qualification [127,128]. Finally, Thomas et al. demonstrated Bayesian inference of the fiber orientation and matrix properties of short fiber-reinforced polymers produced by extrusion deposition additive manufacturing, which is the type of estimation for the fiber architecture that is otherwise missing [76]. These studies show that the estimation methods required for an SFRPC twin are available in neighboring fields; what is missing is their application to the fiber-orientation field of molded parts.

None of the implementations identified in this review (literature covered by the search protocol of Section 1.2, last update October 2026) closes the loop for the SFRPC fiber architecture, that is, automatically updates an estimate of the orientation field of each molded part from process data and adjusts the process settings accordingly. The virtual process chain for sheet molding compound of Görthofer et al. [13] and the FE–DMN method of Gajek et al. [21] provide validated physics-based and surrogate components that such a twin would require, but both are used offline, as digital models. Because the search was not exhaustive, this statement describes the absence of such implementations in the covered literature rather than proof that none exists; unpublished industrial implementations may also exist.

**Table 12:** Representative digital model, digital shadow, and digital twin implementations relevant to composite and polymer manufacturing, classified according to Kritzinger et al. [23]. The integration level was assigned by the authors of this review on the basis of the published descriptions; several of the original papers use the term “digital twin”. The column “Term used in original work” gives the designation chosen by the original authors; the integration criteria are defined in Section 8.2.

Study	Process and Material	Physical Data Used	Digital Components	Integration Level	Term Used in Original Work	Fiber Architecture Included
Görthofer et al. [13]	Compression molding of sheet molding compound	µCT, mechanical tests (validation)	Process simulation → orientation → mean-field homogenization → FE	Digital model	Virtual process chain	Yes (predicted orientation)
Gajek et al. [21]	Injection-molded short fiber-reinforced components	RVE simulations (training)	FE–DMN multiscale surrogate	Digital model (enabling component)	FE–DMN method	Yes (orientation-dependent)
Hürkamp et al. [114]	Overmolding of thermoplastic composites	Production parameters	FE thermal simulation + random-forest surrogate; decision support	Digital shadow	Digital twin	No
Hürkamp et al. [115]	Thermoforming of thermoplastic composite sheets	Process parameters	FE + reduced-order model (POD, decision trees)	Digital model (real-time capable)	Simulation-based digital twin	No
Seon et al. [119]	Autoclave prepreg layup (debulking)	CT void data	CT-informed FE simulation	Digital model	Digital twin	Voids only
Zambal et al. [120]	Composite parts	Manufacturing and inspection data	Effects-of-defects FE analysis	Digital shadow	Digital twin	Defects
Liau et al. [121]; Nasiri et al. [122]	Injection molding	Machine and process sensors	Monitoring, ML, case-based reasoning	Concept or digital shadow	Digital twin	No
Fernández-León et al. [116]	Resin transfer molding of composite preforms	Five cavity-pressure sensors	Deep-learning surrogates trained on synthetic Darcy-flow data; online monitoring	Digital shadow	Digital twin	No (filling state)
Iglesias et al. [123]	Resin transfer molding	Pressure sensors; flow-front observations	Bayesian inversion (ensemble Kalman, sequential Monte Carlo) of permeability	Digital shadow (estimation demonstrated)	Bayesian inversion	No (permeability field)
Yokoyama et al. [124]	Curing of composite laminates	Temperature measurements (numerical study)	Ensemble Kalman filter with cure-kinetics model	Digital shadow (numerical)	Data assimilation	No
Hopmann et al. [126]	Injection molding	Cavity pressure	Iterative learning control of the cavity-pressure profile	Digital twin of process states	Self-optimizing injection molding	No
Thomas et al. [76]	Extrusion deposition additive manufacturing of short fiber-reinforced polymers	Tensile tests	Hierarchical Bayesian inference with micromechanics	Digital model (probabilistic)	Bayesian inference; step toward probabilistic digital twins	Yes (inferred orientation)
Knapp et al. [127]; Mukherjee and DebRoy [128]	Metal additive manufacturing (related field)	Process monitoring (proposed)	Mechanistic heat-transfer and fluid-flow models, sensing, ML	Digital model (building blocks, concept)	Digital twin	Microstructure of metals (predicted)

### 8.3. Proposed Architecture for SFRPCs

**Figure 6:**
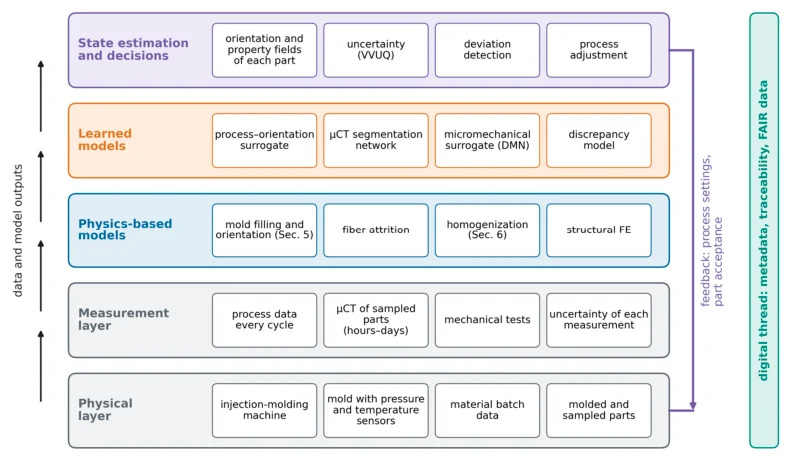
Proposed layered architecture of a Digital Twin for SFRPC injection molding. Process data are acquired at every cycle, and µCT and mechanical data are obtained from sampled parts. The state-estimation layer combines physics-based and learned models with these observations to estimate the fiber-orientation and property fields of each part together with their uncertainty, and feeds decisions back to the process. The digital thread records the metadata that make the data traceable and reusable.

On the basis of Section 2, Section 3, Section 4, Section 5, Section 6 and Section 7, a Digital Twin for SFRPC injection molding can be organized in five layers linked by a digital thread (Figure 6):

1. *Physical layer*: injection-molding machine, mold with cavity-pressure and temperature sensors, material batch data, and sampled parts.
2. *Measurement layer*: process data acquired at every cycle, and offline µCT and mechanical tests of sampled parts at defined intervals, with documented analysis methods and uncertainties (Section 4).
3. *Physics-based model layer*: calibrated mold-filling and orientation simulation (Section 5), fiber attrition model, and homogenization and structural analysis (Section 6).
4. *Learned-model layer*: surrogates for the process–orientation and orientation–property mappings, segmentation networks for µCT data, and discrepancy models that correct systematic simulation errors (Section 7).
5. *State-estimation and decision layer*: estimation of the current orientation and property fields of each part with their uncertainty, detection of deviations, and proposal of process adjustments.

The element that distinguishes this architecture from existing simulation chains is the state-estimation layer. Because the fiber architecture cannot be measured inline, it must be estimated by combining a prior provided by the physics-based model (or its surrogate) with the available observations: cycle-by-cycle process signals and occasional µCT measurements. Bayesian updating and sequential data assimilation provide the formal framework for such estimation, but their application to fiber-orientation fields has not yet been reported in the covered literature; Bayesian inference has so far been applied to global orientation parameters of test specimens rather than to the orientation fields of molded parts [76]. A practical implementation could proceed in stages: first, a digital shadow that predicts the orientation and stiffness fields of each part from its recorded process data using a validated surrogate; second, periodic recalibration of the surrogate whenever µCT measurements of sampled parts become available; and third, closed-loop process adjustment once the prediction uncertainty has been quantified and shown to be sufficiently small for the decisions at hand [24]. Section 8.5, Section 8.6, Section 8.7, Section 8.8 and Section 8.9 position the architecture relative to established reference architectures and specify the estimation methods, the VVUQ framework, the computational requirements, and an illustrative application.

### 8.4. Digital Thread and Data Management

A Digital Twin depends on a digital thread—the linked record of design, process, inspection, and test data across the product life cycle [129]. For SFRPCs, each µCT dataset must be traceable to the material batch, the machine settings, the cycle number, the sampling location, and the analysis method and parameters used to derive the descriptors. Without this metadata, measurements cannot be reused for recalibration or for training ML models. Adopting the FAIR principles—findable, accessible, interoperable, and reusable data [130]—and standard formats for orientation-tensor fields would also enable the multi-laboratory datasets that are currently missing (Section 9).

### 8.5. Positioning Relative to Reference Architectures

The proposed architecture is compatible with established Digital Twin reference architectures but specializes them for SFRPC manufacturing (Table 13). The ISO 23247 series defines a framework for Digital Twins in manufacturing that distinguishes the observable manufacturing elements, a data collection and device control entity, a core entity responsible for operation and management, application and service, and resource access and interchange, a user entity, and cross-system functions such as data assurance and security [131]. Tao et al. described a five-dimensional model consisting of the physical entity, the virtual entity, services, Digital Twin data, and the connections between them [132], and Lu et al. reviewed the enabling technologies of Digital Twin-driven smart manufacturing under a reference model that systematizes their development [133]. The layers of Figure 6 map onto these elements without conflict. The elements that are specifically motivated by SFRPC manufacturing are the following. (i) The main state variable—the fiber architecture—is latent: it cannot be observed inline and must be estimated, which makes a state-estimation layer with explicit uncertainty indispensable rather than optional. (ii) Observations arrive at two very different rates—process signals at every cycle and µCT data from a few sampled parts at intervals of hours to weeks—so that the twin must fuse frequent indirect data with rare direct data. (iii) The model layer is a multiscale chain whose links involve model-form choices (orientation model, closure, homogenization scheme, mapping) that must be documented and versioned together with their parameters. (iv) The twin combines a process twin (machine and mold states) with a material twin of each part in the sense of Kalidindi et al. [31], which operate on different time scales. (v) Uncertainty must be propagated through all layers to support decisions, as required by the VVUQ perspective of the National Academies [24].

### 8.6. State Estimation for the Fiber Architecture

The state-estimation layer combines a forecast of the fiber architecture of each part, provided by the physics-based or learned model for the recorded process data, with the available observations. In a sequential (filtering) formulation, the state vector **x** contains the orientation tensor field—or a reduced representation of it—together with uncertain parameters, such as *C*_I_, and slowly varying process biases; the observation vector **y** contains process signals and, when available, µCT-derived orientation descriptors; and an observation operator *h* maps the state to the observations. For linearized operators and Gaussian errors, the update reads

(9) $$x^{a}=x^{f}+K\left[y-h\left(x^{f}\right)\right],\;\;K=P^{f}H^{T}{\left(HP^{f}H^{T}+R\right)}^{-1}$$

where **x**^f^ and **x**^a^ are the forecast and analysis states, **P**^f^ is the forecast error covariance, **H** the linearized observation operator, and **R** the observation error covariance [134]. Table 14 compares candidate methods for evaluating this update for orientation fields. The Kalman filter (KF) is exact for linear-Gaussian problems, and its extended version (EKF) linearizes nonlinear models; both require the full covariance matrix to be stored and propagated, which is prohibitive for orientation fields with 10^5^–10^7^ degrees of freedom. The ensemble Kalman filter (EnKF) replaces **P**^f^ by the sample covariance of an ensemble of model runs [135]; with ensembles of the order of 50–100 members and covariance localization, it is the most practical option for high-dimensional fields, and it has already been used to estimate the permeability during RTM and the curing heat of composites from sensor data [123,124]. Its cost is dominated by the ensemble of forward simulations, which therefore requires fast surrogates. Particle filters (sequential Monte Carlo) make no Gaussian assumption but suffer from weight degeneracy in high dimensions and are suitable only for low-dimensional states [136]. Variational methods (3D-Var and 4D-Var) compute the state that minimizes a cost function combining the deviations from the prior and from the observations [137]; they require adjoint or differentiable models—which surrogates and PINNs can provide—and are well suited for batch reanalysis when µCT results become available. Bayesian calibration with Markov chain Monte Carlo (MCMC) sampling and a discrepancy term [75] gives the most complete description of uncertainty for a small number of parameters, as demonstrated for the orientation of SFRPC specimens by Thomas et al. [76].

Two properties of orientation fields require special care. First, the analysis must remain admissible: Equation (9) is linear and can produce tensors that are not positive semidefinite or do not have unit trace. Updating a parametrization that guarantees admissibility—for example, eigenvalues mapped through a softmax function together with eigenvector angles, or the parameters of a reconstructed orientation distribution—avoids this problem at the cost of a nonlinear observation operator. Second, the dimension of the state must be reduced. Because orientation fields are strongly correlated in space and are governed by a few process variables, they can be represented by a small number of modes obtained by proper orthogonal decomposition of simulated fields, by the latent variables of an autoencoder, or by the parameters of the orientation model itself; estimation then operates on tens of coefficients rather than on millions of tensor components.

For estimating the orientation field from sparse µCT data, three algorithms are particularly suitable. (i) Model-based Bayesian calibration uses the µCT profiles from a few regions to update the parameters of the orientation model and, if needed, a discrepancy model, after which the calibrated simulation predicts the field everywhere [75,76]; this is the most robust option, because the physics fills the gaps between the measured regions. (ii) Gappy proper orthogonal decomposition reconstructs the full field from a few measured locations by a least-squares fit of the coefficients of POD modes computed from an ensemble of simulations [138]; it is fast enough to be applied to every part but inherits the bias of the simulations used to build the modes. (iii) Gaussian-process (GP) regression of the residual between measured and simulated orientation, with a spatial covariance whose correlation lengths reflect the layered structure, interpolates the correction between measured regions and provides a local uncertainty that grows with the distance from them [139]. These algorithms can be combined: the simulation provides the mean, gappy POD or a GP the correction, and the resulting uncertainty map indicates where the next µCT region should be placed.

Incomplete and noisy process data are handled within the same framework. Measurement noise enters the observation error covariance **R**, which should be estimated from repeated cycles and sensor specifications rather than assumed. Implausible values are rejected by innovation gating, in which an observation is discarded when its deviation from the forecast exceeds a multiple of the predicted standard deviation of the innovation. Missing signals, such as those of a failed cavity-pressure sensor, are handled by removing the corresponding rows of **y**, **H**, and **R**, so that the update uses only the available data and the posterior uncertainty increases accordingly; if critical inputs are missing, the part is flagged rather than estimated. Slowly drifting sensor offsets and batch-to-batch material variations can be included in the state vector as bias terms and estimated together with the fiber architecture. µCT results that become available days after the corresponding parts were molded are assimilated retrospectively with an ensemble smoother, which updates the estimates of past parts and the parameters used for future parts. Finally, every estimate should carry a data-quality flag that records which observations were used, so that decisions can account for incomplete information.

### 8.7. Verification, Validation, Uncertainty Quantification, and Decision Criteria

A practical VVUQ framework for the SFRPC twin can follow the layers of the architecture (Table 15). Verification checks that each code solves its equations correctly: mesh and time-step convergence of the mold-filling and orientation simulation, correct implementation of closures and mapping, and agreement of surrogates with the models they replace on held-out simulations. Validation compares predictions with independent measurements at the level at which decisions are made: orientation profiles from µCT regions not used for calibration, local stiffness from specimens cut from parts, and component responses such as warpage or stiffness. Uncertainty quantification propagates the input, parameter, and model-form uncertainties of Table 8 to the quantities of interest (Section 6.3) and checks that the predicted uncertainties are calibrated, i.e., that the stated 95% intervals contain about 95% of the validation measurements [24]. Validation should be repeated when the material, mold, or machine changes, and the validated domain should be recorded in the digital thread together with the model version.

Whether the uncertainty of the estimated orientation and property fields is small enough for a process adjustment depends on the decision, and it can be stated explicitly with three criteria. First, the prediction intervals must be calibrated in the sense defined above; an uncalibrated uncertainty cannot be used for decisions. Second, a predicted deviation Δ of a quantity of interest from its target—for example, the local modulus in a critical region or the width of the core layer—justifies an adjustment only if it is significant relative to its standard uncertainty *u*(Δ), i.e., |Δ| > *k*·*u*(Δ) with a coverage factor *k* of about 2–3, and only if the uncertainty of the predicted effect of the adjustment is smaller than the tolerance band of the quantity. Third, for acceptance decisions, the posterior probability that a part violates its specification, computed from the estimated distribution of the quantity of interest, should be below an agreed risk level; equivalently, the expected cost of acting should be lower than that of not acting. Until these criteria are met in validation, the twin should operate in a shadow mode in which it proposes adjustments that are reviewed by an engineer, consistent with the human-centric approach of Industry 5.0 [140]; automatic adjustment can then be introduced for well-validated quantities and narrow adjustment ranges.

### 8.8. Computational Cost and Scalability

The architecture must operate on three time scales. Per cycle—typically 10–60 s for injection-molded parts—the twin must record the process data, evaluate the orientation and property fields of the part, and update the state estimate. A full three-dimensional mold-filling and orientation simulation takes minutes to hours and is therefore unsuitable for this task, so that the per-cycle evaluation must rely on surrogates or reduced-order models. The available evidence indicates that this is achievable: neural-network surrogates predicted orientation tensors in seconds instead of hours [18], reduced-order models based on proper orthogonal decomposition provided temperature fields in real time [115], and surrogates based on deep material networks make component-scale nonlinear analyses feasible [21,102]. An ensemble update with 50–100 surrogate evaluations on a reduced state of tens to hundreds of coefficients can be expected to take seconds on current hardware, which is compatible with industrial cycle times. Per batch or shift, recalibration tasks—assimilation of new µCT data, update of discrepancy models, and validation checks—can run within hours on a server or cloud infrastructure without affecting production. Per change in material or mold, the offline generation of training simulations (typically hundreds of runs), µCT campaigns, and surrogate training take days to weeks and dominate the total cost. Scalability across many machines is achieved by sharing the offline models for each material–mold combination and running only the per-cycle evaluation close to the machine (edge computing), while storage, which grows with the number of parts, should be limited to reduced representations of the estimated fields and their uncertainties.

### 8.9. Illustrative Application

To illustrate how the layers interact, Table 16 walks through the architecture for the injection-molded, short glass fiber-reinforced Venturi-shaped part for which µCT data from several regions and calibrated orientation models have been published [19,54]. In the offline phase, the iARD-RPR parameters are calibrated with a Bayesian procedure against µCT profiles of three regions, a surrogate of the filling and orientation simulation is trained for the admissible range of injection speed, melt temperature, and mold temperature, and its validity is checked on held-out simulations and on a fourth µCT region. In production, the cavity-pressure and temperature signals of each cycle are fed to the surrogate, and an ensemble update yields the orientation and stiffness fields of each part with their uncertainty. If a drift in the process signals shifts the predicted modulus in the region of reduced cross-section by more than *k*·*u*(Δ), the twin proposes an adjustment of the injection speed, which is reviewed by an engineer in shadow mode. At regular intervals, a part is sampled for µCT; its measured profiles are assimilated retrospectively, and the parameters and the discrepancy model are updated. The example is not an actual implementation; it shows which data, models, and decisions each layer requires, and it corresponds to a staged implementation in which the digital shadow—prediction from process data—precedes the closed loop.

## 9. Challenges and Future Directions

**Data.** The most important obstacle is the lack of paired datasets that link documented process conditions, spatially resolved µCT measurements of orientation and fiber length, and mechanical properties for the same parts. Published studies typically cover one material, one geometry, and a few processing conditions, and differences in scanning and analysis protocols make them difficult to combine [39,62]. Round-robin comparisons of µCT orientation analysis and open benchmark datasets would benefit both model validation and machine learning. Synthetic annotated datasets, such as the one recently published for fiber-orientation estimation [95], are a useful first step but do not replace measured data.

**Physics-based models.** Orientation models still require material-specific calibration and do not predict clustering, local fiber-content variations, or curvature. Coupled prediction of orientation and fiber length, validated against simultaneous measurements, remains a clear gap [34]. For properties beyond stiffness—strength, fatigue, and failure—the link between orientation tensors and component behavior is weaker, and defects must be considered explicitly [9].

**Uncertainty and validation.** Uncertainty quantification across the chain is largely missing. Priorities are (i) quantifying the measurement uncertainty of µCT-derived descriptors, (ii) determining which model parameters are identifiable from the available data, and (iii) propagating uncertainties to component-level predictions [24,57].

**Artificial intelligence.** ML models trained on simulations reproduce the errors of these simulations, whereas models trained on experiments are limited by small datasets. Network architectures with built-in mechanics such as DMNs [20,21], physics-informed training [104], and transfer learning [97] are promising ways to improve data efficiency and extrapolation. Uncertainty estimates and out-of-distribution detection should become standard outputs of ML models intended to support process decisions.

**Digital Twin implementation.** Moving from digital shadows to digital twins requires state estimation for the fiber architecture, real-time-capable surrogates, interoperable data infrastructures, and a clear definition of the decisions that the twin is intended to support. Industrial frameworks that emphasize human oversight and resilience, such as the Industry 5.0 concept [140], are consistent with a staged approach in which the twin first supports engineering decisions before it is trusted with automatic process adjustment.

Table 17 condenses these challenges into a research roadmap that identifies priority research actions, the research and industrial communities that need to be involved, and an indicative time horizon for advancing SFRPC Digital Twins from digital models and digital shadows toward validated and, eventually, closed-loop implementations.

## 10. Conclusions

This review has followed the fiber architecture of short fiber-reinforced polymer composites from its formation during processing to its representation in Digital Twins. The main conclusions are as follows:

1. The fiber architecture—above all the orientation and length distributions—is the state variable that links processing to properties. Because it varies within each part and through its thickness, it must be treated as a field rather than as a material constant.
2. X-ray µCT has become the reference method for three-dimensional characterization; individual glass fibers can be analyzed at voxel sizes of about 1 µm. The resolution–volume trade-off and the dependence of the results on the analysis algorithm mean that µCT data should be reported with their analysis parameters and uncertainties, and used primarily for calibration and validation.
3. Orientation models have evolved from the Folgar–Tucker equation to formulations with slow kinetics (RSC, RPR), anisotropic rotary diffusion (ARD, iARD), and flow–fiber coupling through orientation-dependent viscosity (IISO). They agree better with measurements after calibration but remain phenomenological, and the choice of closure approximation and mesh also affects the results.
4. Mean-field homogenization combined with orientation averaging is mature for stiffness predictions; predictions of strength and fatigue, mapping between meshes, and uncertainty propagation are less developed.
5. Artificial intelligence has delivered its clearest benefits in fiber segmentation and in micromechanical surrogates with built-in physics. Process–orientation surrogates are limited by their training data, and physics-informed learning has not yet been applied to orientation evolution in mold filling, although physics-informed closure models for homogeneous flows have recently appeared (Section 7.4).
6. Existing Digital Twin implementations for composite and polymer manufacturing are digital models or digital shadows; none (within the literature covered by our search protocol) closes the loop for the fiber architecture. A layered architecture with an explicit state-estimation layer is proposed, together with a staged implementation path. Paired process–microstructure–property datasets and systematic uncertainty quantification are the main prerequisites for its realization.

Looking forward, five actions would move SFRPC Digital Twins from digital models and digital shadows toward validated closed-loop systems (Table 17): (1) establish open, paired datasets with documented µCT analysis protocols and uncertainties; (2) report orientation-model parameters with posterior uncertainties and use measured or fitted fourth-order tensors for stiffness predictions; (3) validate ML models with group-wise splits along the axes of material, geometry, machine, and process conditions, and correct simulation bias with discrepancy or multi-fidelity models; (4) demonstrate state estimation of orientation fields, first as a digital shadow on industrial parts, using ensemble or variational methods with surrogates; and (5) define decision criteria based on calibrated uncertainty and operate the twin in shadow mode before allowing automatic process adjustment.

## Figures and Tables

**Table 1:** Scope of representative reviews and integrative studies compared with the present review. ✓, main focus; (✓), partly covered; –, not a focus. The assessment was made by the authors of this review.

Reference	Type and Focus	Process and Orientation	µCT	Multiscale Modeling	AI/ML	Digital Twin	Fiber Architecture as DT State
Tucker [5]	Monograph; description, measurement, and prediction of fiber orientation	✓	(✓)	(✓)	–	–	–
Kugler et al. [12]	Review; fiber orientation models	✓	–	–	–	–	–
Baum et al. [29]	Review; numerical simulation of the filling phase in injection molding	(✓)	–	–	–	–	–
Garcea et al. [14]	Review; X-ray CT of polymer composites	–	✓	(✓)	–	–	–
Maire and Withers [25]	Review; quantitative X-ray tomography	–	✓	(✓)	–	–	–
Görthofer et al. [13]	Integrative study; validated virtual process chain of sheet molding compound	✓	✓	✓	–	–	(✓)
Sharma et al. [30]	Review; machine learning for polymer composite materials	–	–	(✓)	✓	–	–
Malashin et al. [26]	Review; machine learning for composite process optimization	(✓)	–	–	✓	–	–
Jones et al. [27]; Tao et al. [28]	Reviews; Digital Twins in manufacturing and industry	–	–	–	(✓)	✓	–
National Academies [24]	Report; foundational research gaps of Digital Twins, VVUQ	–	–	–	(✓)	✓	–
Kalidindi et al. [31]	Perspective; Digital Twins for materials	–	(✓)	✓	(✓)	✓	(✓)
Present review	Critical review; full process–structure–property chain of SFRPCs	✓	✓	✓	✓	✓	✓

**Table 2:** Literature search protocol. Terms within a block were combined with OR, and each thematic block was combined with the material block M using AND.

Block	Topic	Search Terms (Title, Abstract, Keywords)	Time Coverage
M	Material	“short fiber*” OR “short fibre*” OR “discontinuous fiber*” OR “fiber-reinforced thermoplastic*” OR “long fiber thermoplastic*” OR “sheet molding compound”	No restriction
A	Orientation formation and modeling	“fiber orientation” AND (“injection molding” OR “compression molding”) AND (model* OR simulation OR “Folgar–Tucker” OR “rotary diffusion” OR closure)	No restriction
B	Characterization	(“micro-computed tomography” OR µCT OR “X-ray tomography” OR “optical microscopy”) AND (“fiber orientation” OR “fiber length” OR segmentation)	No restriction
C	Multiscale modeling	(homogenization OR “Mori–Tanaka” OR “mean-field” OR “orientation averaging” OR “representative volume element”) AND (stiffness OR “process chain” OR “integrative simulation”)	No restriction
D	AI/ML	(“machine learning” OR “deep learning” OR “neural network*” OR “physics-informed” OR surrogate) AND (composite* OR “injection molding”)	2018–2026
E	Digital Twins	(“digital twin*” OR “digital shadow” OR “data assimilation” OR “Bayesian calibration”) AND (composite* OR “injection molding” OR “polymer processing”)	2018–2026; definitions and standards without restriction
–	Complementary	Backward and forward citation searches from key papers; Google Scholar for proceedings and reports	Last update October 2026

**Table 4:** Main processing and material parameters affecting fiber orientation in injection molding (compiled from [4,5,34,56]).

Parameter	Primary Effect on Flow	Typical Effect on Orientation	Consequence for the Part
Injection speed	Shear rate; frozen-layer growth	Wider shell and narrower core at higher speed (material-dependent)	Through-thickness anisotropy
Melt temperature	Viscosity	Modifies shell-to-core ratio via viscosity and frozen layer	Anisotropy, shrinkage
Mold temperature	Frozen-layer growth	Extent of the skin layer and of locked-in orientation	Surface properties, warpage
Gate location and geometry	Flow type (diverging, converging)	Transverse core in diverging flow; local disorder near gate	Local anisotropy, warpage
Part geometry (ribs, holes, thickness changes)	Local flow kinematics	Strong in-plane and three-dimensional variations; weld lines	Local weak zones, stress concentrations
Fiber content	Interaction intensity	Lower steady-state alignment; slower kinetics	Higher stiffness, lower predictability
Fiber length and aspect ratio	Rotational dynamics; attrition	Stronger alignment, but more breakage	Reinforcement efficiency

**Table 6:** Comparison of fiber orientation model families.

Model	Key Idea	Parameters	Strengths	Limitations
Jeffery [46]	Hydrodynamic rotation of isolated rigid fibers	Aspect ratio	Exact for dilute suspensions	No interactions; overestimates alignment at industrial concentrations
Folgar–Tucker [47]	Isotropic rotary diffusion due to interactions	*C*_I_	Simple, robust, widely implemented	Too-fast kinetics; core width underestimated
RSC [49]	Objective retardation of eigenvalue growth	*C*_I_, *κ*	Captures slow kinetics	Additional parameter; closure dependence
ARD-RSC [48]	Anisotropic diffusion tensor combined with RSC	Five ARD coefficients, *κ*	Accurate for short and long fibers after fitting	Parameter identification difficult
iARD-RPR [70,71]	Two-parameter anisotropic diffusion with retarding principal rate	*C*_I_, *C*_M_, *α*	Few parameters; improved core-width prediction	Material-specific calibration
IISO viscosity coupling [53,73]	Orientation-dependent scalar viscosity (flow–fiber coupling)	Coupling parameters	Captures dominant coupling at moderate cost	Not an orientation model; additional calibration
Length–orientation coupling [34]	Attrition model solved with orientation	Breakage parameters	Predicts length-distribution field	Limited validation data

**Table 7:** Mean-field homogenization schemes used for SFRPCs.

Scheme	Principle	Typical Use	Strengths	Limitations
Mori–Tanaka, two-step [11,77,78]	Fibers embedded in the average matrix strain; aligned estimate averaged with Equation (5)	Stiffness of injection-molded parts; default in most software	Explicit and robust; coincides with the lower Hashin–Shtrikman–Walpole bound for aligned fibers	Underestimates stiffness at high fiber contents; one-step version can give non-symmetric tensors for misaligned fibers
Tandon–Weng [79]	Closed-form Mori–Tanaka solution for aligned spheroids	Aligned step of the two-step procedure	Explicit engineering constants	Elastic and aligned only; isotropic constituents
Lielens (interpolative double inclusion) [83,84]	Interpolation between Mori–Tanaka and inverse Mori–Tanaka with a weight depending on fiber volume fraction	Higher fiber contents; nonlinear analyses	Good agreement with experiments and full-field data in several comparisons [11,84]	Interpolation weight is empirical
Incremental Mori–Tanaka (tangent, secant, second-moment) [82,85]	Linearized matrix behavior at each load increment	Plasticity, viscoplasticity, damage	Component-scale nonlinear analyses at moderate cost	Too stiff tangent predictions without corrections; matrix model must be calibrated
Full-field RVE (FE, FFT) [63,86]	Explicit microstructure with fiber ends and contacts	Reference solutions; strength; clusters	Resolves local fields	Computational cost; RVE size
Deep material network [20,21]	Trained hierarchy of laminate building blocks	Nonlinear component analyses	Close to full-field accuracy at much lower cost	Training required for each microstructure class

**Table 13:** Correspondence between established Digital Twin reference architectures and the layers of the proposed SFRPC architecture.

Proposed Layer (Figure 6)	ISO 23247 [131]	Five-Dimensional Model [132]	SFRPC-Specific Requirement
Physical layer	Observable manufacturing elements	Physical entity	Machine, mold, and material batch; each molded part is an instance with its own fiber architecture
Measurement layer	Data collection and device control entity	Digital Twin data; connections	Two-rate data: cycle-wise process signals and sparse, delayed µCT and test data with documented uncertainty
Physics-based model layer	Core entity (digital modeling and representation)	Virtual entity	Multiscale chain with model-form choices (orientation model, closure, homogenization, mapping)
Learned-model layer	Core entity (digital modeling and representation)	Virtual entity; services	Surrogates with documented validity range; discrepancy models that correct simulation bias
State-estimation and decision layer	Core entity (operation and management; application and service); user entity	Services	Latent orientation field estimated with uncertainty; decision rules based on calibrated uncertainty
Digital thread	Resource access and interchange; cross-system functions	Digital Twin data; connections	Traceability of µCT data to batch, cycle, location, and analysis method

**Table 14:** Candidate state-estimation methods for the fiber-orientation field of SFRPC parts.

Method	Principle	Feasibility for Orientation Fields	Main Limitations	Suggested Use in the SFRPC Twin
KF, EKF [134]	Linear(ized) Gaussian update with full covariance	Only for reduced states (up to about 10^3^ variables)	Covariance storage; linearization errors; admissibility of tensors not guaranteed	Update of a few POD coefficients or model parameters
EnKF and ensemble smoother [123,135]	Covariance approximated by an ensemble of model runs	Feasible for large fields with 50–100 members and localization	Requires fast surrogates; sampling errors; Gaussian assumption	Cycle-wise update from process data; retrospective assimilation of delayed µCT data
Particle filter (sequential Monte Carlo) [136]	Weighted samples; no Gaussian assumption	Low-dimensional states only	Weight degeneracy in high dimensions	Discrete states (e.g., material batch, model selection)
3D-Var, 4D-Var [137]	Minimization of prior and observation misfit	Feasible with differentiable surrogates or adjoint models	Adjoint development; uncertainty only approximate	Batch reanalysis after µCT measurements
Bayesian calibration (MCMC) with discrepancy [75,76]	Posterior distribution of parameters and model discrepancy	Low-dimensional parameter vectors	Sampling cost; identifiability of discrepancy and parameters	Calibration of orientation-model parameters from µCT and tests
Gappy POD [138]	Least-squares fit of POD modes to sparse data	Fast for any field size	Inherits the bias of the simulated modes; mode truncation	Reconstruction of the full field from a few µCT regions
Gaussian-process regression [139]	Probabilistic interpolation of residuals	Moderate data sizes (up to about 10^4^ points)	Choice of covariance function; scaling	Local correction and uncertainty maps; planning of µCT locations

**Table 15:** Practical VVUQ framework for an SFRPC Digital Twin.

Layer or Element	Verification	Validation	Uncertainty Quantification	Example Acceptance Criterion
Filling and orientation simulation	Mesh and time-step convergence; benchmark flows	µCT profiles from regions not used for calibration; short shots for front positions	Parameter posteriors; comparison of model forms	About 95% of measured points within the predicted 95% intervals
Closure and homogenization	Comparison with exact or measured fourth-order tensors; full-field RVE references	Stiffness of specimens cut in different directions and locations	Closure and scheme treated as model-form uncertainty	Deviation within the combined uncertainty
Mapping	Preservation of tensor properties; comparison of fields before and after mapping	–	Interpolation error	Admissible tensors at all integration points
Learned models	Agreement with parent model on held-out simulations; group-wise splits	Predictions for new parts, molds, or materials	Ensembles or Bayesian networks; out-of-distribution detection	Group-wise test error below the tolerance of the quantity
State estimation	Synthetic twin experiments with known truth	Retrospective comparison with µCT of sampled parts	Posterior covariance; innovation statistics	Normalized innovations consistent with the assumed covariances
Decision layer	Logic and interface tests	Shadow-mode comparison of proposed and engineering decisions	Probability of nonconformance	|Δ| > *k*·*u*(Δ), *k* ≈ 2–3; risk below the agreed level

**Table 16:** Illustrative walk-through of the proposed architecture for an injection-molded short glass fiber-reinforced Venturi-shaped part (based on the published data of refs. [19,54]; procedures are indicative).

Layer	Offline (Before Production)	Per Cycle	Per Batch or Sampling Interval
Physical	Mold with cavity-pressure and temperature sensors; material batch records	Molding of part *n*	Sampling of parts for µCT and mechanical tests
Measurement	µCT of three regions (calibration) and of a fourth region (validation)	Cavity-pressure and temperature curves; machine set-points	µCT orientation profiles of sampled parts (available after days)
Physics-based model	Filling and iARD-RPR orientation simulation; Bayesian calibration; mean-field homogenization	–	Additional simulations if the process window changes
Learned model	Surrogate trained on calibrated simulations; validity checks	Surrogate evaluation (seconds)	Update of the discrepancy model with new µCT data
State estimation and decision	Prior ensemble of fields and parameters	Ensemble update of orientation and stiffness fields; test of |Δ| > *k*·*u*(Δ); proposal reviewed by an engineer	Retrospective smoothing; parameter update; validation report
Digital thread	Calibration data, model versions, and validity range recorded	Part identifier linked to cycle data and estimate	µCT data linked to batch, cycle, location, and analysis method

**Table 17:** Research roadmap for SFRPC Digital Twins.

Unresolved Challenge	Priority Research Actions	Communities Involved	Horizon
Paired process–microstructure–property data	Open benchmark datasets with documented process data, µCT of several regions, and mechanical tests; FAIR metadata for orientation-tensor fields	µCT and testing laboratories; molders and material suppliers; data-infrastructure initiatives	Short term (1–3 years)
Measurement uncertainty of µCT descriptors	Round-robin analyses; phantoms and synthetic ground truth; multi-reference validation of segmentation; reporting of voxel size, algorithm, and averaging volume	µCT and image-analysis community; standardization bodies	Short term
Identifiable and uncertain orientation-model parameters	Bayesian calibration with discrepancy models; identifiability analyses; parameter databases with uncertainties	Rheology and process-simulation community; software vendors	Short to medium term
Model-form errors (closure, coupling, length)	Comparisons with measured fourth-order tensors; coupled orientation–length models; flow–fiber coupling in three-dimensional flows	Process modeling and micromechanics	Medium term (3–5 years)
Uncertainty propagation to the component	Hierarchical uncertainty quantification across the chain; spatially correlated error models; validation at component level	Computational mechanics; uncertainty-quantification community	Medium term
Transferable ML models	Group-wise validation protocols; multi-fidelity and transfer learning; learned closures and discrepancy models; operator learning	Machine learning and scientific computing; process modeling	Medium term
State estimation of orientation fields	Admissible reduced parametrizations; ensemble and variational methods with surrogates; demonstration as digital shadow on industrial parts	Data assimilation and control engineering; industry	Medium term
Closed-loop operation and trust	Decision criteria with calibrated uncertainty; shadow-mode trials; standard-compliant architectures (ISO 23247); human oversight	Manufacturing industry; standardization bodies; Industry 5.0 initiatives	Long term (more than 5 years)

## Data Availability

No new data were created or analyzed in this study. Data sharing is not applicable to this article. The illustrative calculation in Section 6.3 uses only the input values listed in the notes to Table 9.

## Abbreviations

The following abbreviations are used in this manuscript:

3D-Var, 4D-Var	Three- and four-dimensional variational data assimilation
AI	Artificial intelligence
ANN	Artificial neural network
ARD	Anisotropic rotary diffusion
CNN	Convolutional neural network
CT	Computed tomography
DL	Deep learning
DMN	Deep material network
DT	Digital Twin
EKF	Extended Kalman filter
EnKF	Ensemble Kalman filter
FE	Finite element
FFT	Fast Fourier transform
GP	Gaussian process
IBOF	Invariant-based optimal fitting (closure)
iARD	Improved anisotropic rotary diffusion
IFSS	Interfacial shear strength
IISO	Informed isotropic (viscosity model)
KF	Kalman filter
LSTM	Long short-term memory
MCMC	Markov chain Monte Carlo
ML	Machine learning
µCT	X-ray micro-computed tomography
MT	Mori–Tanaka
PINN	Physics-informed neural network
POD	Proper orthogonal decomposition
ROI	Region of interest
ROM	Reduced-order model
RPR	Retarding principal rate
RSC	Reduced-strain closure
RTM	Resin transfer molding
RVE	Representative volume element
SFRPC	Short fiber-reinforced polymer composite
VVUQ	Verification, validation, and uncertainty quantification
